# Applications of Artificial Intelligence in the Health Sector: A PRISMA-Based Systematic Review

**DOI:** 10.3390/healthcare14162604

**Published:** 2026-08-19

**Authors:** Zakir Hossen Shaikh, Sarita Yadav, Bibhu Prasad Sahoo, Jay Shankar Sharma, Abdelrhman Meero

**Affiliations:** 1 Department of Finance and Accounting, College of Business Administration, Kingdom University, Riffa P.O. Box 40434, Bahrain; z.shaikh@ku.edu.bh (Z.H.S.); ar.meero@ku.edu.bh (A.M.); 2 Amity School of Economics, Amity University Haryana, Gurugram 122413, India; 3 Department of Commerce, SGTB Khalsa College, University of Delhi, Delhi 110007, India; bpsahoo@sgtbkhalsa.du.ac.in; 4 Department of Commerce, PGDAV College (Evening), University of Delhi, Delhi 110065, India; jayshankarsharma@pgdave.du.ac.in

**Keywords:** artificial intelligence, health, artificial intelligence in healthcare, digital health, ethics, bibliometric analysis, PRISMA, digital health applications

## Abstract

**Highlights:**

**What are the main findings?**

- The health sector has benefitted from artificial intelligence (AI), with the increasing focus on data-centric approaches like machine learning, electronic health records (EHRs), and digital health systems. Most of the research on theme AI in healthcare focuses on technical and clinical applications, with themes of data governance, ethics, and health equity having fewer focus points and being less central.
- The publication trends reveal that the Global North is advancing in research on AI segments. China is exceptionally leading from the Global South because of its world-class infrastructure and huge R&D in the health sector.

**What are the implications of the main findings?**

- AI’s influence on health research has witnessed a boom during the post-COVID era, with many research organizations and governments acknowledging significant potential in the field, yet there remains a huge potential that has not been reaped.
- Lack of attention on data regulation is one of the flaws, indicating the need for better policy integration globally to tackle data privacy, transparency and ensure accountability. AI is acting as a revolutionary tool in the healthcare system, but ethical considerations are a must to ensure its long-term legal usage.

**Abstract:**

**Background:** The health sector is getting transformed with the usage of AI, be it diagnosis, treatment planning, disease prediction, and or health system management. Research in this field has picked up in the last few years, which was made possible with the emergence of machine learning, natural language processing and the increasing number of e-health records. **Objectives:** The study aims to investigate the current trends in the implementation of artificial intelligence (AI) applications in medical settings by investigating the global scientific output/landscape on this theme, such as the annual publication trends, country-wise contributions, and publishing patterns. **Methods:** The current study is based on systematic review by combining bibliometric analysis and cluster analysis using VOSviewer version 1.6.20, R software version 4.5.0, and Biblioshiny (Bibliometrix package in R) along with preferred reporting items for systematic reviews and meta analyses (PRISMA), 2020 which provides transparency and rigorous visualization to examine the articles published in English on the use of AI in healthcare, after the onset of COVID-19 till date i.e., from 2020 to 2026 on the Scopus database. **Results:** Using the relevant search string, 5940 documents were identified between 2020 and 2026, 1434 were included for analysis after screening and relevant filters. The publications have increased remarkably after 2020 on this theme and more than half of the publications have their roots in the discipline of Medicine. The USA, China, and the United Kingdom have contributed the most to the volume of research. Natural language processing and diagnosis are the emerging themes. The Journal of Medical Internet Research, BMC Medical Informatics and Decision Making, Computers in Biology and Medicine, IEEE Journal of Biomedical and Health Informatics, Frontiers in Public Health, and Digital Health are some of the most influential sources in the field. Li J and Liu X are among the authors with remarkable local impact. **Conclusions:** The work aims to assist investigators, health care professionals, and policymakers to learn about modern trends and focus on critical areas of future research and collaboration in AI-enhanced health care. The limitation of the study is that it considered only the Scopus database but it has opened up opportunities for researchers for analysis using other databases such as Dimensions, Lens, and PubMed. Also, this review is considering the publication record since the onset of COVID-19 but a comparative analysis of pre and post-pandemic studies can also be conducted to get a holistic view of drastic collaboration of research in this field. **Discussions:** The findings suggest that the role of artificial intelligence in health care has paramount over recent years, with other supporting technologies but a technologically hesitant population as well as low acceptance of AI due to ethical issues, cannot be ignored for ensuring efficiency in the health sector.

## 1. Introduction

**Figure 1:**
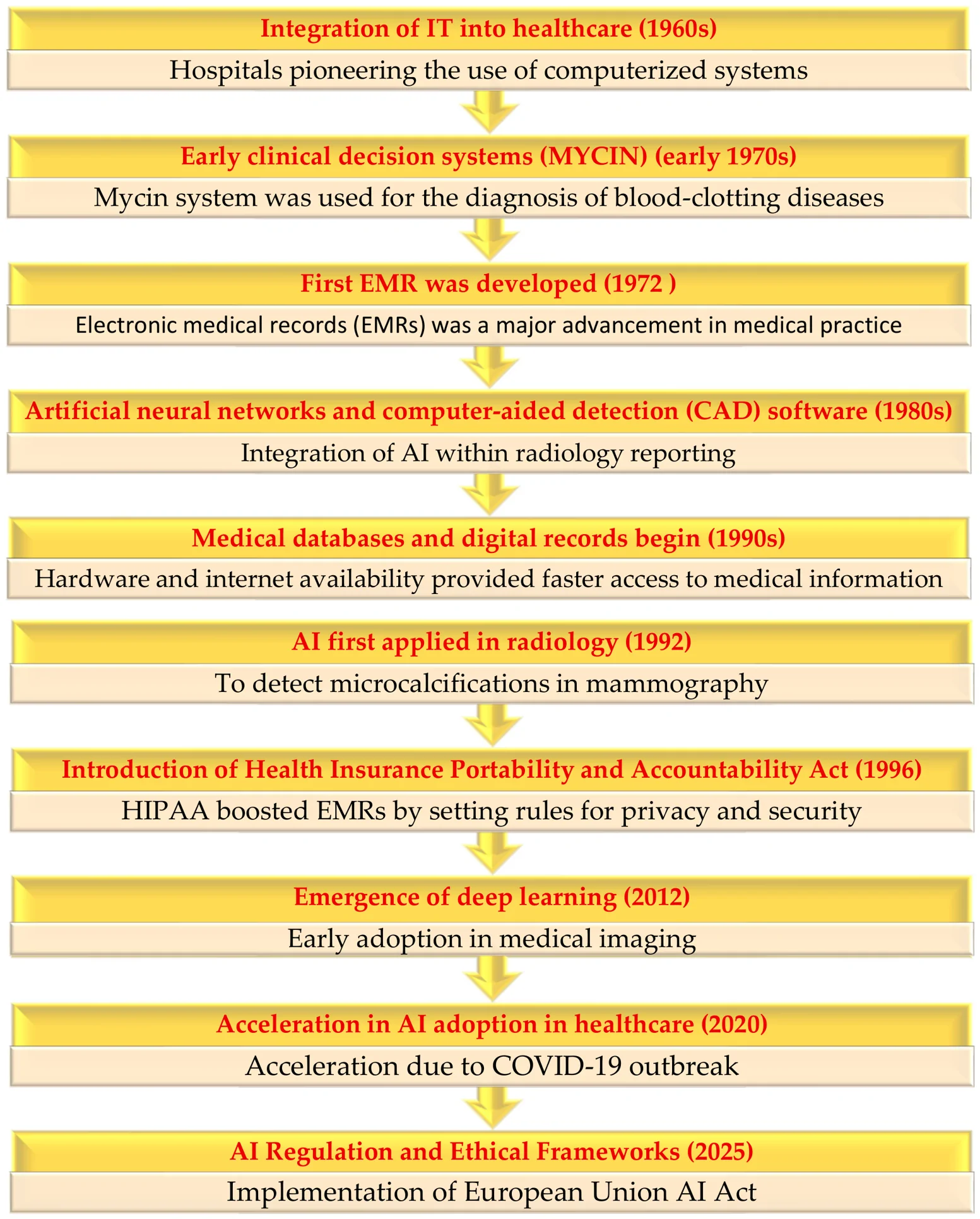
Timeline of AI and digitalization in the health sector. Source: Authors own elaboration.

Artificial Intelligence gained importance in health care services recently, especially after arrival of the COVID-19 pandemic, that peaked the need for advanced and personalized treatment to optimize efficiency of the health delivery systems using big data analytics. Health care industry is transforming with assistance of AI, which allows analyzing vast amounts of data on patients [1,2,3]. Health care providers can use AI systems to design individualized treatment plans that can be specific to each patient through analyzing personalized patient data and genetic patterns to support precision medicine. This individualistic therapy increases diagnostic accuracy, allows the early diagnosis of diseases, and improves the outcomes of treatment over the traditional one-size-fits-all treatment of the human body [4]. Additionally, AI-based systems can memorize and get acquainted with the past diagnostic and treatment results, forming an ever-growing body of knowledge. Such gathered data may be recycled to aid in diagnosis and treatment of other patients with similar illnesses or health-related problems and hence enhance clinical consistency and minimize human error. Unlike traditional approaches to treatment procedures, which may involve numerous laboratory studies and lengthy diagnostic tests, AI-based technologies can simplify the diagnostic process, save time, money, and relieve patients and their families from financial obligations [5]. The complex patient data, i.e., the data related to the symptoms, the medical history, and genetic markers, can also be processed with high accuracy using AI technologies. Through this analysis, AI systems are able to provide alternative treatment options, recommend the right medications and forecast the possible risks or side effects of certain treatments. The predictive ability enables medical workers to make better decisions and respond to potential complications both during and after therapy [6]. Altogether, this field has a substantial potential to transform the health care delivery, making it more efficient, cost-effective, and facilitating decision-making of health care professionals based on the available data. In spite of the obstacles concerning data privacy, ethical concerns, and regulations, AI is still one of the most effective tools in the development of contemporary health care systems. An in-depth review will help to know the current scenario and future scope of this theme. The foundation of it can be the timeline of remarkable technical interruptions in health sector (Figure 1).

Schwedler et al. (2026) [7] argue that telemedicine gained importance recently, especially during the pandemic. But the rapid deployment of AI during COVID-19 raised questions about validation and generalizability of models applied using limited or biased datasets. Hussain et al. (2024) [8] argue that this instant AI adoption during recent years lacks robustness and has created risks for long-term sustainability and trust.

The paper goes on as follows: objectives are well defined along with research questions and suitable tools to be used for achieving the objectives; the methodology section provides essential details of bibliometric methods and PRISMA 2020. The conclusion section discusses findings, followed by limitations and policy recommendations that concludes the paper and provides the future scope of the research.

### 1.1. Literature Review

A few bibliometric studies have been conducted that see the impact of AI on the health sector but most of the studies have a narrowed focus on major health issues, leading to a scope for further bibliometric analysis. Exploratory research needs to be done so that the most relevant and productive authors, affiliations and sources related to the field can be identified, which will help researchers to identify suitable information with less effort. This research will assist policy makers and health care specialists to inculcate the latest and advanced AI-integrated techniques into the health sector.
**Author (Year)****Title****Objectives****Methodology****Findings****Research Gaps**Amanda Yee et al. (2026) [9]“Family caregivers’ acceptance of Artificial Intelligence-enabled technologies for providing care to older adults”To examine acceptance of AI-enabled technologies for cardiogram diagnosis by older adults and aged.Cross-sectional survey of 199 respondents who needed cardiogram diagnosis and are aged 45+ who have digital access.Out of 9 variables studied, 6 displayed a positive attitude toward AI-enabled technologies. Huge costs, trust in the source of advice for care and technology anxiety stop respondents from relying on AI-enabled treatment.Privacy concerns and effective solution to reduce burden of caregivers were not paid enough attention. Respondents from a single firm raise question of sampling bias, which has ended the role of some demographic variables.Amel Dawod Kamel Gouda et al. (2026) [10]“Empowering nursing students during AI era: educational strategies for enhancing knowledge and acceptance of artificial intelligence”To know the effect of participation in 10-session program among nursing undergraduates.Using responses of 1000 nursing students from Sohag University (Egypt), a pre-test and a post-test were conducted. Pearson correlations and Analysis of Covariance (ANCOVA) analysis were made.Technology acceptance model (TAM) shows consistency in results. The findings identified 64.7% correlation in program delivery on AI-integration to nursing students and improvement in knowledge.Limited evidence on improved clinical competency after AI training. Also, the study was carried out at a single institution.Schwedler et al. (2026) [7]“The Role of Telemedicine Centers and Digital Health Applications in Home Care: Challenges and Opportunities for Family Caregivers”To evaluate the acceptance of telemedicine centers in home care and their role in lives of patients’ families. Through PRISMA analysis various relevant studies were identified from various databases.Responses were collected from 58 family caregivers, alongside a case study was conducted.There is very little acceptance of telemedicine, so there is need for improvement in the level of public trust in technology. Digital health applications have the potential to make home care effective. The present scenario can be improved with effective implementation of telemedicine in home care and continuous organizational support.The study does limited discussion on understanding of digital literacy barriers faced by caregivers. Generalization becomes difficult with a sample size of 58. Also, the results are based on self-reported information this may raise question on reliability of the findings.Ke Zhang et al. (2026) [11]“Optimization of academic performance and mental health in college students through an AI-driven personalized physical exercise and mindfulness intervention system”To examine the effect of AI-driven personalized intervention on academics and mental wellbeing among university students in eastern China.328 undergraduate students from three universities comprised a control group to study AI-personalized interventions, standardized interventions, and controls.Customized interference of AI improves academic performance by 10.28%, reduces 36.7% stress, and helps in 28.4% physiological improvement than the control case.Lacks comparison with traditional interventions and human-led programs. Convenience sampling may lead to biased results.Shrika Vejandla et al. (2026) [12]“Impacts of cognitive forcing and need for cognition on biased AI assisted decision making about mental health emergencies”To know how AI-impacted decisions influence Cognitive force (CF), and need for cognition (NFC) related to violence risk.Violence risk of 281 respondents was observed to determine the effect of AI recommendations on CF and NFC. Then 373 other responses were randomized.Cognitive force (CF) interventions have led to increased bias because these are influenced by stereotypes, whereas NFC interventions that are influenced by psychological factors have noticed a reduced biasness of suggestions provided by AI.The study lacks discussion on AI bias mitigation strategies and lacks testing of ethical accountability.Suriá-Martínez et al. (2026) [13]“Artificial Intelligence and Emotional Support: A Comparative Study of University Students with and Without Disabilities”To evaluate the role of AI- based technologies in providing emotional support among students with and without disabilities.A sample of university students aged 18 to 30 was chosen, out of which 88 participants had some disability, while 270 had no disability. A questionnaire with three dimensions: AI familiarity, frequency of AI use, and perceived usefulness of AI was ranked on a 5-point Likert scale.The results indicated that students without disabilities were familiar with the technology compared to the disabled. This highlights attention requirements for disabled students at university level to promote effective inclusion.This study did not differentiate on basis of types of disabilities. It considers only university students, ignoring youth of other educational levels as well as other age groups and AI emotional-support systems can be included.Yousefi et al. (2026) [14]“Integrating Artificial Intelligence (AI) in Primary Health Care (PHC) Systems: A Framework-Guided Comparative Qualitative Study”The study explores the determinants that influence AI readiness in primary health care in Canada and Iran.Semi-structured interviews and focus group discussions were conducted.Coordination across governance, financing, infrastructure, and workforce is required for smooth use of AI in primary health care.External constraints impacting PHC across countries have been ignored. Study is subject to selection bias as purposive sampling technique has been used. Also, there has been lack of evidence on AI readiness in low-resource PHC systems.Jiangtao Dong et al. (2026) [15]“Attitudes of psychiatric nurses towards the integration of artificial intelligence applications to clinical care: a qualitative study in China”To examine the impact of AI system intervention on students’ academic performance and mental health.This study employed interviews with 15 psychiatric nurses recruited from Henan Mental Health Center and a thematic analysis was conducted using NVivo.Results showed that AI-based personalized intervention in physical habits and thought processes will enhance the overall well-being of individuals, but raised privacy issues.Limited quantitative validation of findings and study does not talk much about improving AI competency of nurses. Also, studying the attitudes of 15 nurses from a tertiary psychiatric hospital makes generalizability difficult.Ashton and Chatfield (2026) [16]“AI-Enhanced Qualitative Analysis in Healthcare: Unlocking Insight from Interviews of Leadership at Top-Performing Academic Medical Centers”To assess how freely large language model (LLM) AI tools can make proper use of healthcare data.Semi-structured interviews were conducted with chief nursing officers and results were analyzed using text mining using latent semantic analysis and Gemini 2.5, Scholar AI 5.1, Copilot with chat 4.0, and Claude’s Sonnet 4.5.LLM AI tools are boon to healthcare settings as these tools have ability to analyze hidden text efficiently and this does not require technical expertise.Data was only collected from chief nursing officers, which limits the scope of study. Limited transparency regarding AI reliability in qualitative coding, and study lacks ethical guidelines for AI use in healthcare.Cihan Unal & Selim Sahin (2026) [17]“Health sciences students’ attitudes toward artificial intelligence: predictors of ethical awareness, clinical decision-making, and public health perceptions- a cross- sectional study”To know acceptance of AI among scholars of health sciences for ethical usage, and appropriate problem-solving.A cross-sectional study of 668 students was conducted, and logistic regression, TAM and Unified Theory of Acceptance and Use of Technology, UTAUT, were applied on the responses.The study found a moderately positive attitude of students towards AI. A mean score of 4.05 for AI is beneficial, along with a mean of 2.52 mentioning risk reduces acceptance of AI in health sector.Collection of data from a university makes generalizability of the findings difficult. The data were collected through self-reporting, which may introduce response bias.Gonçalves et al. (2026) [18]“Impact of Artificial Intelligence on the Care of Terminally Ill Patients”This study tries to explore the impact of AI on palliative care.Using databases such as PUBMED, CINAHL and Web of Science, the study reviews publications from 2015 to 2025.AI in palliative care could help professionals with symptom control and development of communication skills, as well.Limited evidence on patient-centered outcomes; lack of ethical impact assessment on sensitive issues like end-of-life care.Sabat Sani et al. (2026) [19]Adoption of artificial intelligence in primary health care: systematic synthesis of stakeholder perspectives To analyze stakeholder perception on AI adoption.Systematic synthesis using studies from PubMed, Scopus, Web of Science, CINAHL, and grey literature from 2015 to 2025.Six themes were identified- Barriers, Facilitators, Impact on Healthcare Delivery, Ethical/Legal/Social Implications, Stakeholder Perspectives, and Future Directions.Lack of quantitative validation.Yue Zhang (2026) [20]“The value for money of artificial intelligence-empowered precision medicine: a systematic review and regression analysis”To evaluate the price of AI-empowered precision medicine (AI-PM).Systematic review of 48 documents and statistical analysis of 31 documents published from 2013 to 2023 was carried out from various databases.AI-enabled precision medicine can reduce cost and improve non-monetary benefits for patients as well.Lack of standardized economic evaluation models and quantitative analysis was limited to studies using disability-adjusted life-years.Majdi Abdellatief and Raed Shujaa Alotaibi (2025) [21]“From Intelligence to Trust: Evaluating AI-Powered Service Quality for User Satisfaction and Continuance in mHealth”To evaluate how AI-supported mobile health influences satiation and consistency in usage intention in mobile health systems.Using PLS-SEM study investigates how AI impacts service quality, trust, level of satiation and consistent use.AI-enabled service improves user satisfaction (β = 0.753), but fails to bring consistency in usage (β = 0.165). Only users’ satisfaction brings consistency in usage of AI systems, thus this field requires work on improving users’ satisfaction.The study is limited by outdated AI service-quality measures and single cultural settings. Insufficient focus on data privacy, and explainable AI in mHealth adoption is there.Bastami et al. (2025) [22]Artificial intelligence in healthcare: applications, challenges, and future directions. A narrative review informed by international, multidisciplinary expertiseTo provide historical evolution, applications, and implications for health professions of AI.Structured review in Ovid MEDLINE from 2018 to 2025.AI enhances diagnostics, treatment planning, and disease prediction.Lack of standardized clinical validation.Chougule et al. (2025) [1]Artificial Intelligence in Healthcare: A Comprehensive Review of Applications, Challenges, and Future DirectionsTo create ethical frameworks for AI deployment and to analyze AI’s strengths, limitations, and potential in healthcareReview based on AI models such as ANN, XGBoost, CNN, RNN, GENTRL.Algorithmic bias, transparency, and data privacy issues hamper integration of AI in clinical practice. Biased datasets worsen health disparities, and raise trust issues.The study lacks focus on implementation barriers and focus remains on ethical concerns.Chukwurah et al. (2025) [2]Emerging AI Applications in Healthcare: A Comprehensive ReviewTo explore emerging AI tools in healthcare systems.Systematic review.AI tools enhance predictive analytics, early disease detection, and customized treatment but ethical concerns raise the need for robust validation frameworks for equity in implementation of AI technologies in healthcare.The study lacks discussion on governance frameworks.Bekbolatova et al. (2024) [23]Transformative Potential of AI in Healthcare: Definitions, Applications, and Navigating the Ethical Landscape and Public PerspectivesTo examine public perception of AI.Review.AI systems support healthcare professionals by automating routine tasks. But legal and ethical challenges are obstacles and leave a huge scope for AI usage in health sector.Puts less stress on current scenario of AI in health care.Rahman et al. (2024) [24]Impact of Artificial Intelligence (AI) Technology in Healthcare Sector: A Critical Evaluation of Both Sides of the CoinTo evaluate the benefits and risks of AI.Critical review using Scopus-indexed, Web of Science and PubMed databases.AI assists in providing effective treatment but maintaining data privacy, and arranging bias-free data is difficult.Lack of long-lasting impact.Khan et al. (2023) [25]Drawbacks of Artificial Intelligence and Their Potential Solutions in the Healthcare SectorTo analyze drawbacks and risks of AI systems in health sector and solutions to these risks.Review.There is need to develop a complete universal AI based on trustworthy algorithms to improve AI adoption in healthcare.Less stress on how to frame standard guidelines for the moral use of AI in healthcare.Ali et al. (2023) [26]A systematic literature review of artificial intelligence in the healthcare sector: Benefits, challenges, methodologies, and functionalitiesTo analyze benefits, challenges, and applications of AISystematic review using IEEE database, ACM Digital database, Scopus database, Emerald database, Science Direct database, and AIS e-library.AI improves human life quality, efficiency, and decision-making.Real-world deployment issues were not given due importance.

### 1.2. Objectives of the Study

An in-depth analysis of the association between the technological world and the health sector has been conducted by identifying the research developments in the field to date and the future research potential. The study examines the growth in publications, country-wise contributions, and conducts cluster analyses of documents published in the field. This work is a contemporary topic because of its increasing relevance with every passing day. So, the contribution of the study would be better as an updated synthesis of the latest developments. It would be helpful to add clarification from other bibliometric studies with cluster analysis, as well as the PRISMA framework, and make it more obvious how this time frame and data set are different.

### 1.3. Mapping of Research Questions with Research Techniques

Based on these research questions, the research objectives have been designed to validate the crucial role of AI in the health sector:

1. To identify the most influential authors and sources in AI and health care.
2. To identify the annual growth of publications, country-wise distribution, and the most influential countries adding to the literature on AI and health care.
3. To analyze the integration of source collaboration patterns.
4. To explore the frequently used keywords.

**Research Questions****Research Tools/Techniques**RQ1. What is the present status of research keeping artificial intelligence in health sector at the center?Annual scientific production, Life cycle of scientific productionRQ2. Which themes are dominating in this field?Thematic map and factorial mapRQ3. Which countries and affiliations are most productive and influential in publications of AI in health care?Countries and affiliated scientific production and citation analysisRQ4. Which authors and sources are the most influential in this field over the recent years?Author productivity as well as their local impact, and key sources.

## 2. Methodology

This systematic review was conducted in accordance with the preferred reporting items for systematic reviews and meta-analyses (PRISMA 2020) and follows the guidelines for transparent and comprehensive reporting. The Scopus database was searched between 2020 and 2026 to discover current research patterns and future scope of the theme [27]. According to the Scopus website, the database contains 80 million documents, 234,000 books, 7000 publishers, and 80,000 institution records, making it one of the top priorities for bibliometric analysis (Elsevier, 2022 [28]).

VOSviewer is effective in visualizing clusters, hence were used for the same, whereas Biblioshiny has been used for tabular and statistical analysis of bibliometric data [29]. This paper provides an extensive description of the integration of AI in the health sector, thus it seems to throw light on the advancements made with the introduction of AI in health-related services. With the spread of the contagious disease COVID-19 worldwide, its severity added to the need for immediate advancements in health care with the least interference by humans. This incident led to an exponential boom to the world of AI in the health sector, making it the most suitable time period to analyze research published under this theme.

No special control was exercised on self-citations or on duplicate publications since the data were drawn from a standardized Scopus database, which is subject to indexing and duplicate filtering procedures. The review process includes clearly defined eligibility criteria, systematic database searching, study selection, data extraction, and synthesis of findings. In order to promote transparency and reduce potential bias, the study protocol was retrospectively registered in the Open Science Framework (OSF). The registration provides a permanent record of the research plan, including the intended methodology and analysis approach, prior to completion of the analysis. Any deviations from the original protocol, if applicable, are reported and justified within the manuscript.

This study has made use of a multi-layered bibliometric approach that integrates artificial intelligence techniques, healthcare domains, clinical applications, and system-level implementations. Whereas earlier studies lack integration between AI and clinical workflow. Also, the study combines quantitative bibliometric mapping with qualitative network analysis of the relevant studies to identify emerging themes, research gaps, and future directions. The study does not solely rely on bibliometric analysis but also employs an in-depth qualitative synthesis of influential studies using the PRISMA technique. The previous bibliometric studies focus on specific medical domains such as oral health or diagnosis, whereas this study offers a comprehensive, post-COVID, and holistic examination of AI applications in healthcare, integrating bibliometric mapping with qualitative insights on concerns like ethics, regulation, data privacy issues, algorithmic bias, and the divide in AI adoption across the world.

### 2.1. Search Strategy

**Figure 2:**
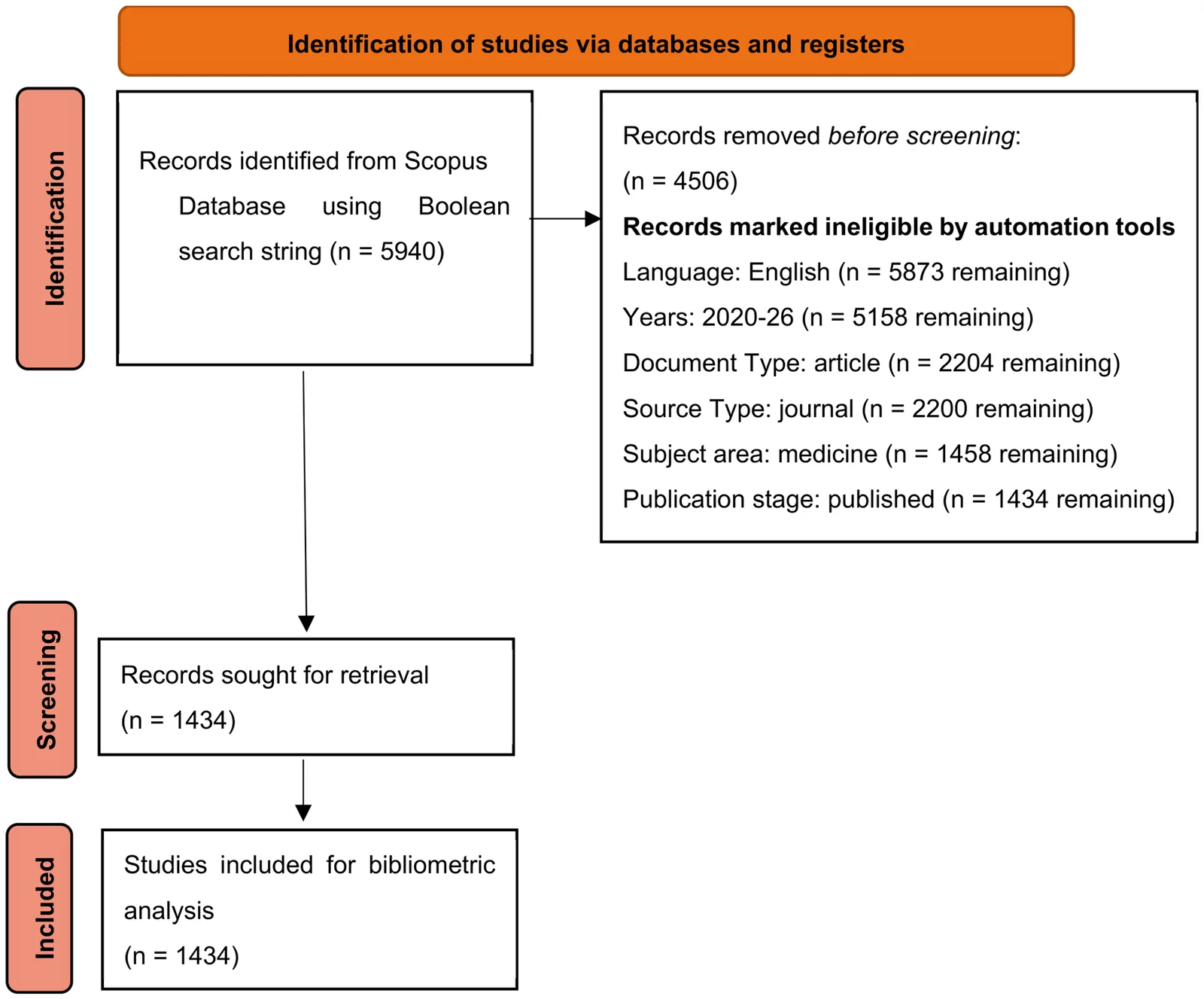
Prisma chart showing inclusion and exclusion criteria of documents for study.

A comprehensive search was conducted on the Scopus database using a Boolean search technique with “AND” and “OR” Boolean operators to select suitable documents [30]. “AND” provides interconnection between the two different topics, whereas “OR” expands the search of documents. In order to get more concise results, the search was applied only for the article title, i.e., excluding keywords and abstracts that are commonly included in searches for bibliometric information to get more concise and related work to the theme (refer to Table 1). A multi-dimensional search string of four layers has been used to search relevant documents. Layer 1 is for AI technologies, level 2 is for the healthcare domain, the next level is for terms related to diagnosis, and the last level focuses on implementation of AI tools in healthcare. This filter of including only the article title restricts the search for retrieving crucial studies, ensuring coverage of relevant studies only. The search strategy included terms related to artificial intelligence and health care as:

The PRISMA flow diagram in Figure 2 reflects the search strategy along with various inclusion and exclusion criteria, applied on documents retrieved in title, abstract, and keywords search field using search (“Artificial intelligence” OR “machine learning” OR “deep learning”) AND (“healthcare” OR “health care”) AND (diagnos*) AND (“decision support system*” OR “electronic health record*”). After getting 5940 records from Scopus database of the documents having the relevant search string mentioned in Table 1 in their title, abstract and or keywords, records were identified on the basis of the English language, then records of publications published from 2020 to 2026 were included. Only research articles were included, as conference papers, book chapters, non-English published work and articles that did not relate to the study were excluded because they could not be compared with the other documents or were irrelevant for this study. The criteria were developed with great care to ensure consistency, meaning and comparability of the data being analyzed. On basis of source type, records published in journals were included. To ensure parsimony and relevance, the analysis was restricted to subject area of Medicine only to capture the intersection of artificial intelligence and healthcare. Finally, records under publication were removed and 1434 articles were chosen for analysis.

### 2.2. Bias Assessment and Quality Evaluation

The methodological quality and risk of bias of the included studies were assessed using a structured evaluation approach appropriate for systematic literature reviews. Each study was independently screened and evaluated based on predefined inclusion criteria and study characteristics such as clarity of objectives, appropriateness of methodology, and transparency of reporting.

Source bias in SLR studies is mainly due to the selection of databases, the language filter in the database and the dynamics of citations. A structured search strategy mentioned in Table 1 was used to search the literature comprehensively from Scopus. The methodological quality of the selected articles was assessed indirectly through bibliometric indicators like number of citations, average number of citations per year, the h-index, and other impact metrics.

What makes this study unique from existing ones is that it uses a combination of PRISMA 2020–directed review with a very recent (2020–2026) Scopus dataset, providing both methodological rigor and updated insights. Unlike a purely bibliometric study whose focus remains on publication trends and citations, this paper provides a detailed and critical synthesis of AI applications, challenges, and policy implications in healthcare, making it a more holistic and practical study.

Table 2 provides relevant details, such as 1434 documents were analyzed from 622 sources published during 2020–2026 and each article has about 15 citations. About 9958 authors have authored these documents and almost all were authored in co-authorship; the average number of co-authors per document is about 8, which shows a good amount of author collaboration to author something productive and about half of the co-authorships were international. This shows that authors from different countries are collaborating on this theme.

**Figure 3:**
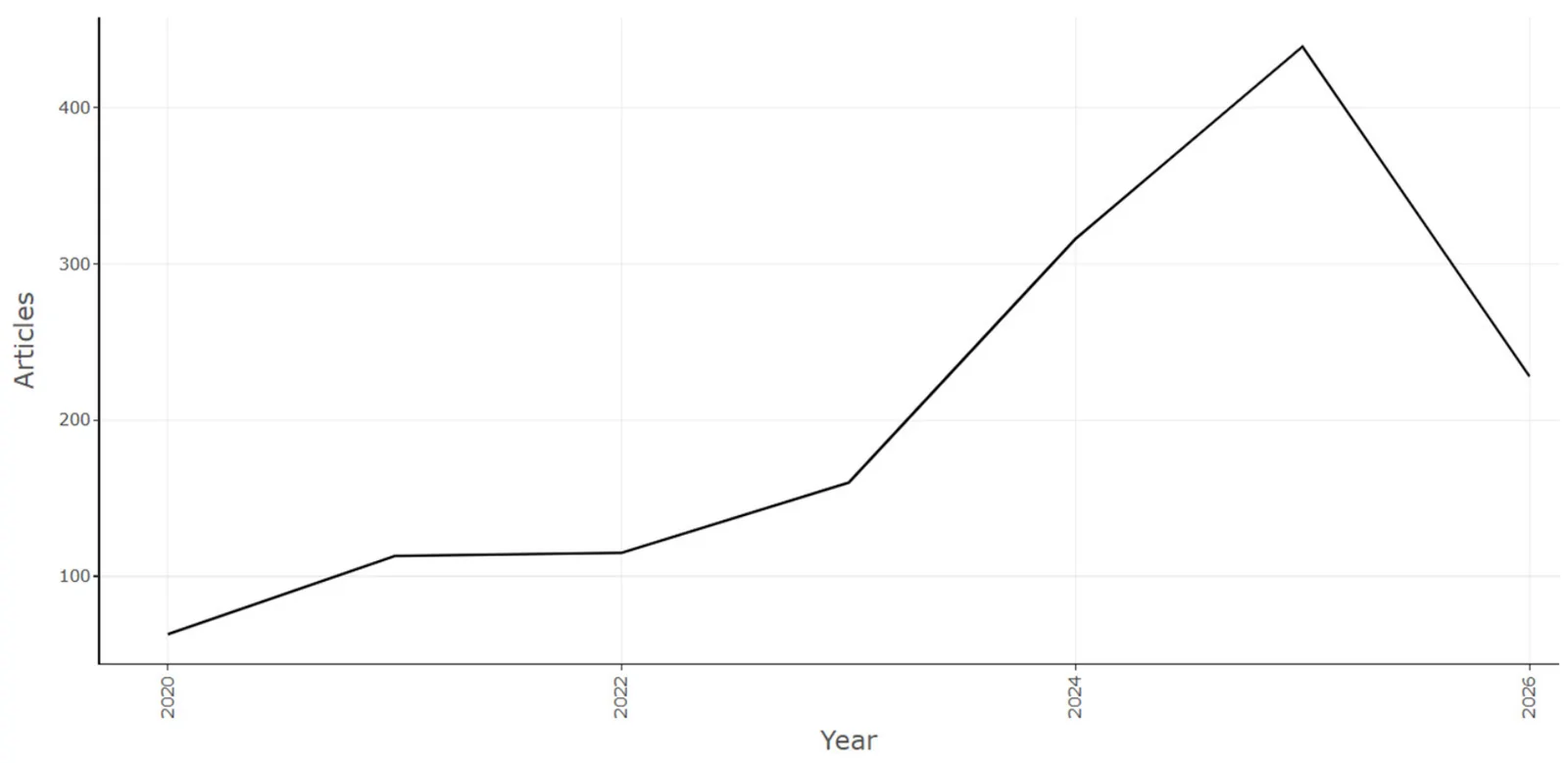
Annual scientific production.

Year by year the articles published on AI and health have been increasing gradually from 63 articles in the year 2020 to more than 400 articles in 2025 and till 2026, more than 200 articles have already been published, showing the rising interest of researchers in this field as reflected by the line graph in Figure 3. The rapid rise in such a short span shows that the theme is well influenced by COVID-19, which demanded sudden advancements in the health sector.

**Figure 4:**
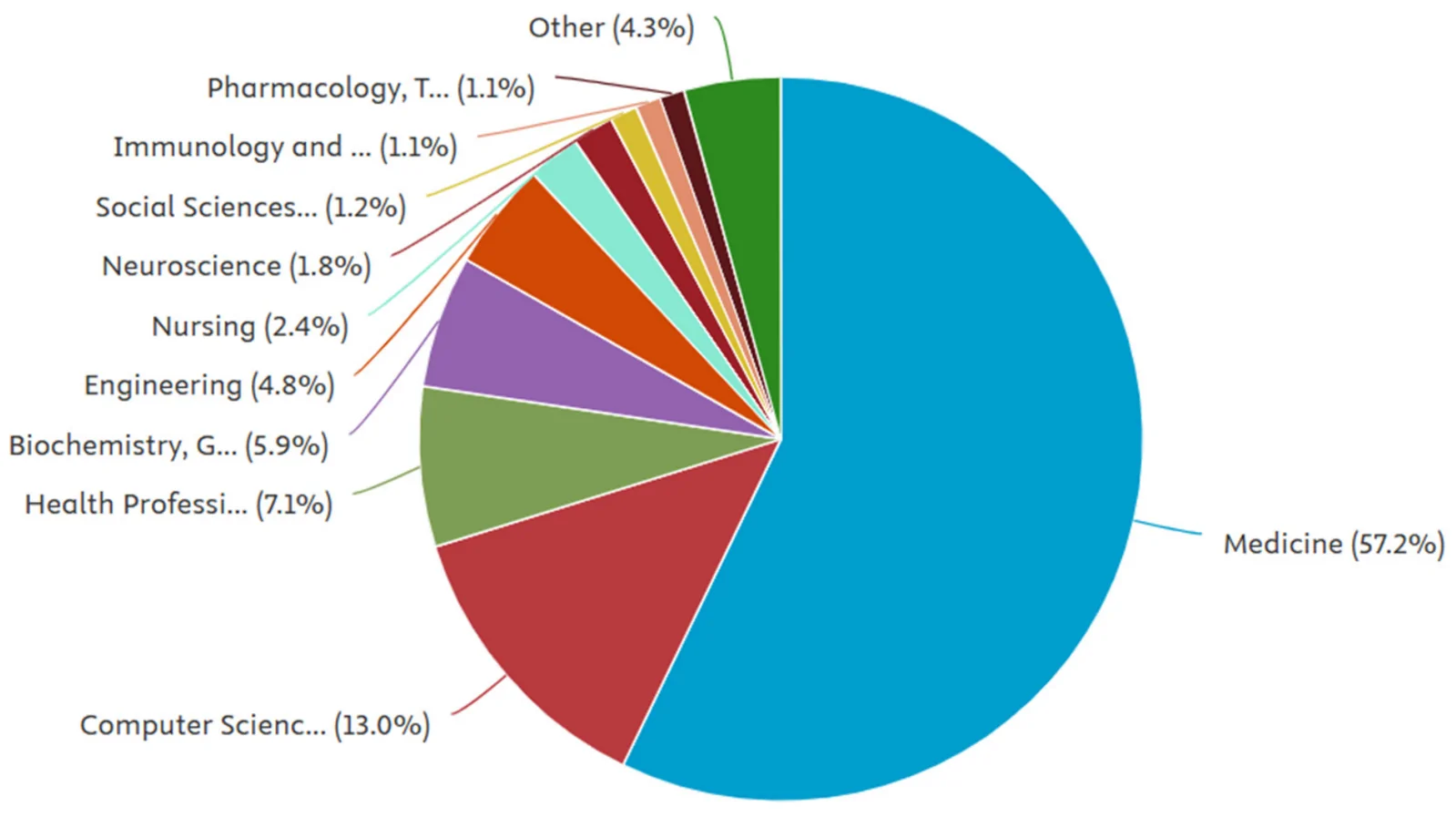
Documents by subject area before subject filter.

Figure 4 shows that major portion of research in this field is from medicine, health professionals and nursing, i.e., the medical field, which is why are included in the analysis.

Table 3 reflects the citation trends on the topic. In 2020, average citations per article as well as average citations per year were highest over the selected years. The number of publications (column N) has been increasing continuously year by year. This trend is in this manner because documents published in earlier years had higher citable years compared to the records published in recent years. Unusually, high average citations for 2020 (57.03 per document) and for 2021 (34.81), but there has been a steep decline in 2025–2026. This may be due to the large number of publications issued at the start of the COVID-19 pandemic, which manifested a high number of cited publications. Recent studies have a shorter citation time period, which means that recently published documents have limited time to accumulate citations.

**Figure 5:**
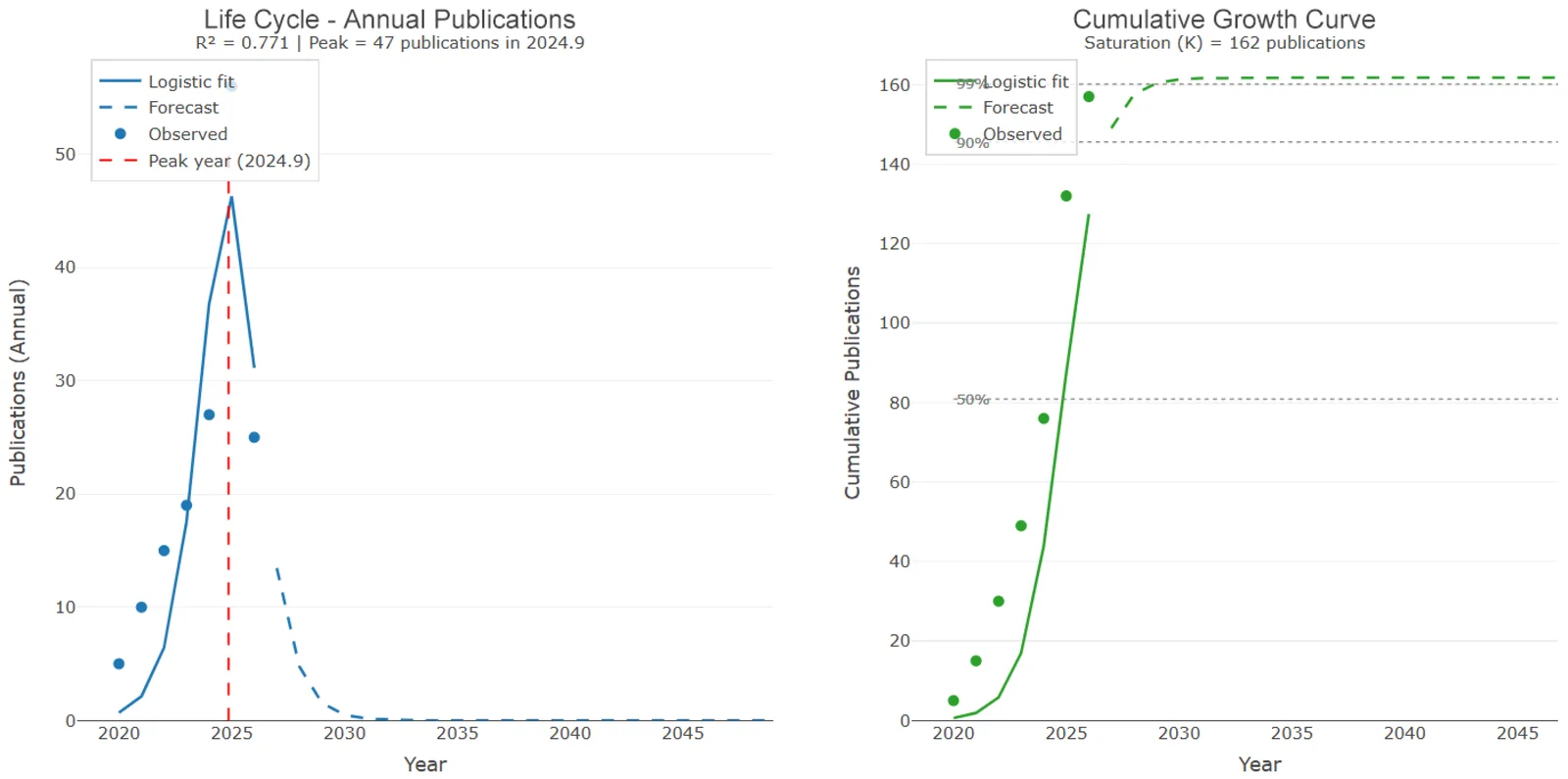
Life cycle of scientific production. Source: Generated from Biblioshiny.

Figure 5 shows life-cycle analysis of annual publications in the left plot, and of cumulative publications on the right plot, based on a logistic growth model to analyze the growth pattern of publications. In the annual publications plot, dots show the actual publications, whereas the line shows the logistic fit. There has been a rapid growth in publications since 2021, reflecting accelerated research, mainly driven by increased interest and demand during the COVID-19 period, and a peak was reached in 2025 with about 390 publications. After the peak curve is expected to fall, reflecting maturity phase of the life cycle of research. The plot on the right seems S-shaped. There has been an increase in the cumulative published work over the initial years. Then with around 1500 publications, the rate of growth in accumulation of publications is estimated to stabilize.

**Figure 6:**
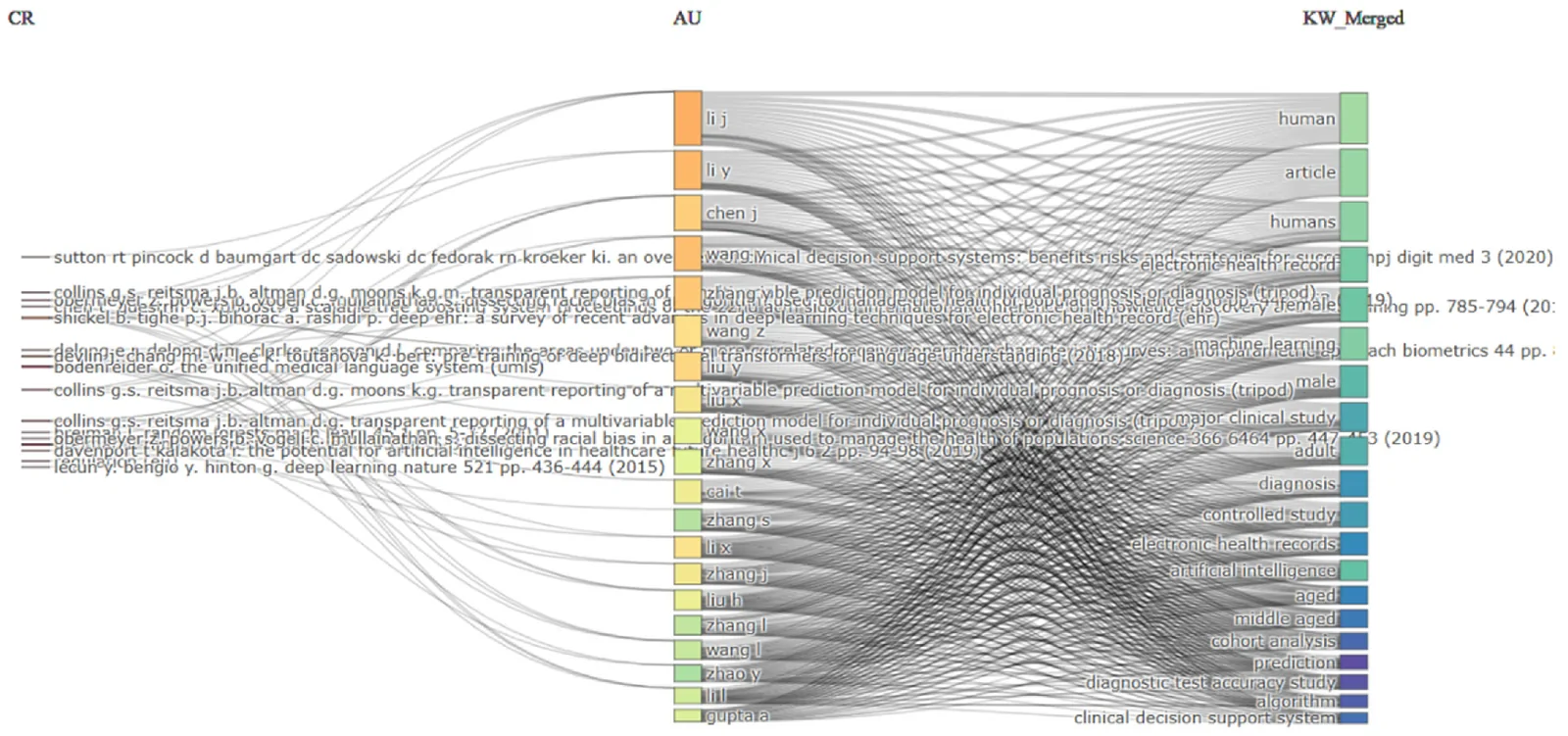
Sankey plot. Source: Generated from Biblioshiny.

Figure 6 shows the association between cited references (CRs), authors (AUs), and merged keywords (KW_Merged). There are fewer key references on the left, which show the most influential and frequently cited studies that have been mapped to the most productive authors, and the mapping of authors and keywords is very dense, reflecting their frequent usage by multiple authors. The strings between these three fields reflect interconnectedness among the fields.

**Figure 7:**
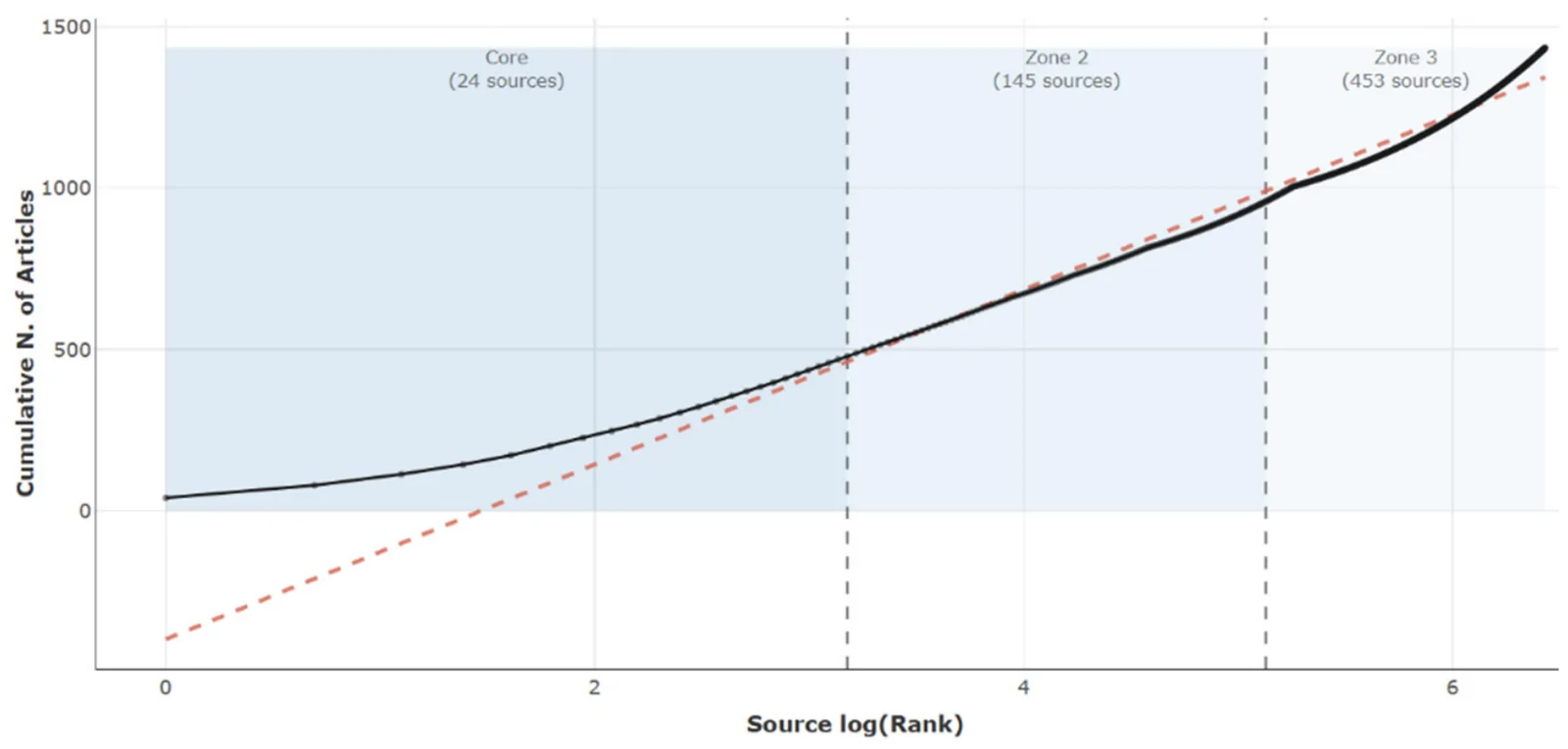
Nucleus from Bradford’s law of scattering. Source: Generated from Biblioshiny.

Bradford’s law has been used to identify foundational sources of foundational knowledge after applying relevant filters, as shown in Figure 7. The plot has been generated by computing the logarithmic rank of each journal. The core journals publish the major share of publications on the topic. All the sources (here only journals) have been classified into three zones based on number of articles published. There are 24 core sources in zone 1 reflecting concentration of articles in these sources, 145 journals in zone 2 and 453 in zone 3, which demonstrates higher number of journals, but each contributes fewer articles. The red dotted line is the estimated one and the actual scenario is well aligned with it. The figure concludes that few journals dominate the field, while a large number of peripheral sources contribute to its expansion.

Table 4 shows the influence of journals within the selected dataset, not globally. It helps identify the most relevant sources that are frequently cited, and form core of the research area based on h-index (h) to assess productivity and citation impact, g-index (g) to identify the top-cited papers, m-index (m) to provide fair comparison among new researchers and senior researchers, total number of citations (TC), total number of publications (NP), and year of start of publication (PY). The Journal of Medical Internet Research performs best among the chosen sources in all the parameters.

**Figure 8:**
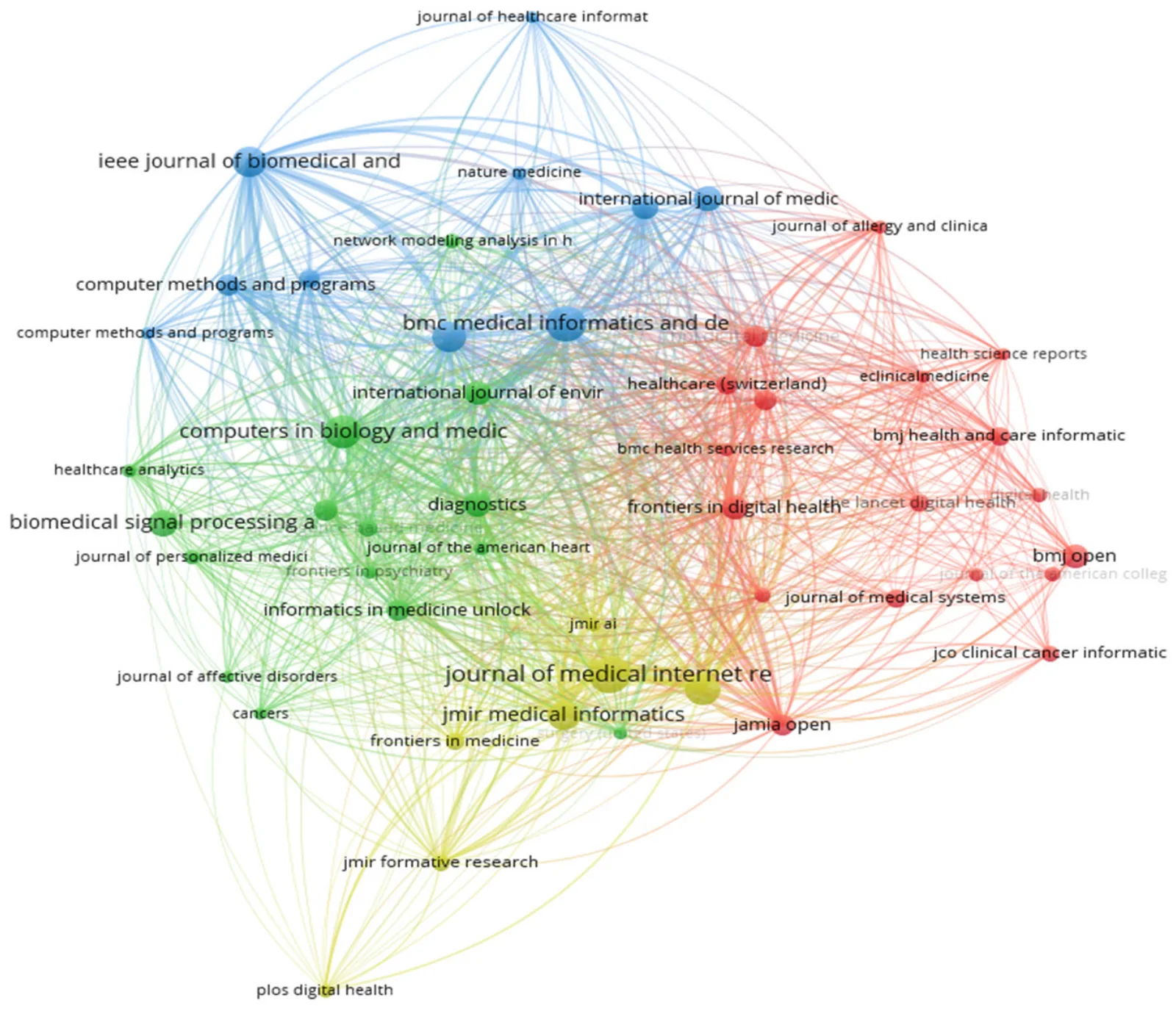
Co-linking of publication sources. Source: Generated using Vosviewer.

This Figure 8 is network analysis of 622 sources of publications out of 50 sources that met the threshold of at least five documents with at least five citations per source, to ensure a wide range of impactful sources. Sources such as BMC Medical Informatics and Decision Making, IEEE Journal of Biomedical and Health Informatics, Computers in Biology and Medicine, and Journal of Medical Internet Research, Frontiers in Public Health, Digital Health are some of the sources from where suitable work related to the field can be found.

**Figure 9:**
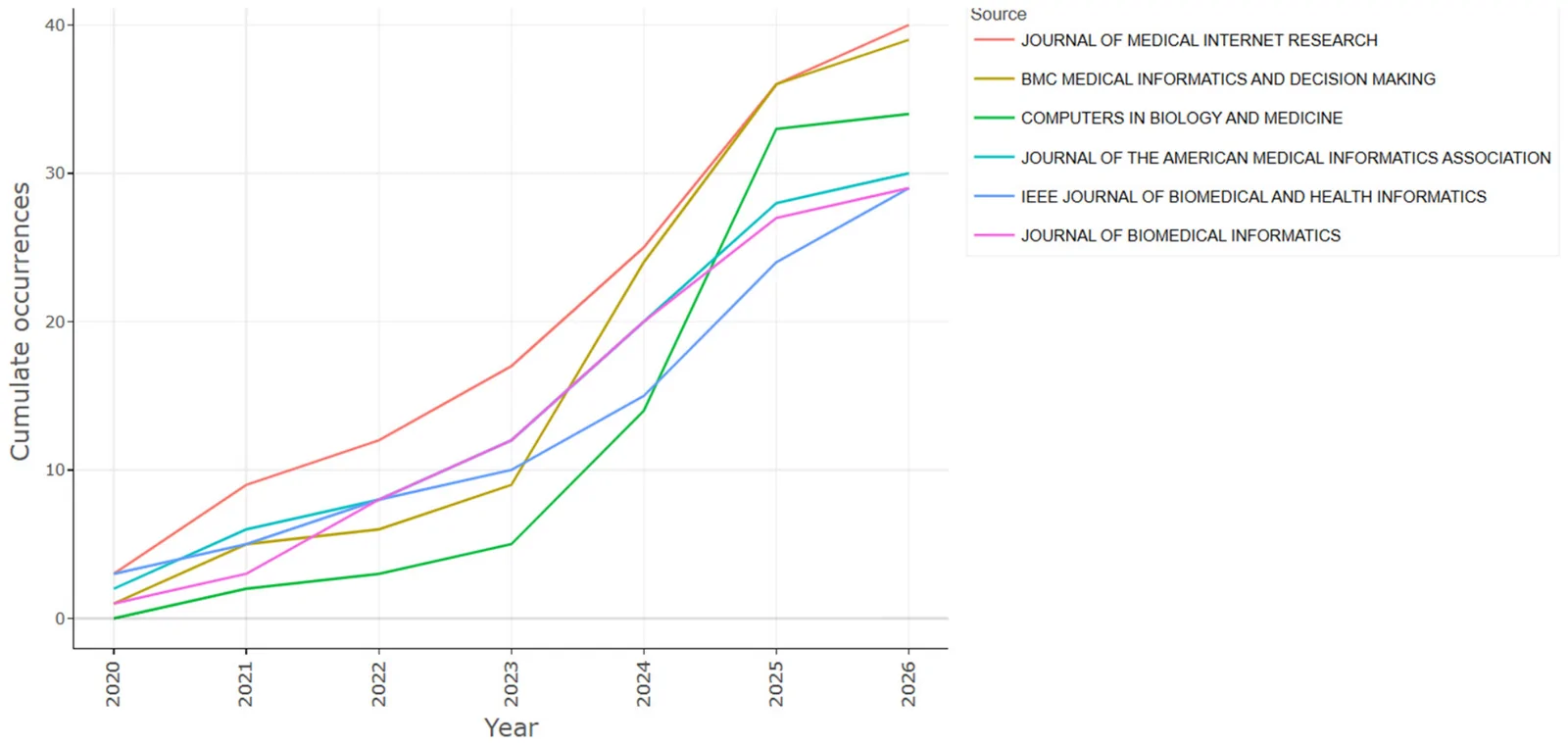
Sources’ production over time. Source: Generated from Biblioshiny.

The annual growth trends of leading journals have been demonstrated in Figure 9. The Journal of Medical Internet Research source has the maximum cumulative documents related to the theme, followed by BMC Medical Informatics and Decision Making. Computers in Biology and Medicine showed a rapid increment in publications after 2023.

**Figure 10:**
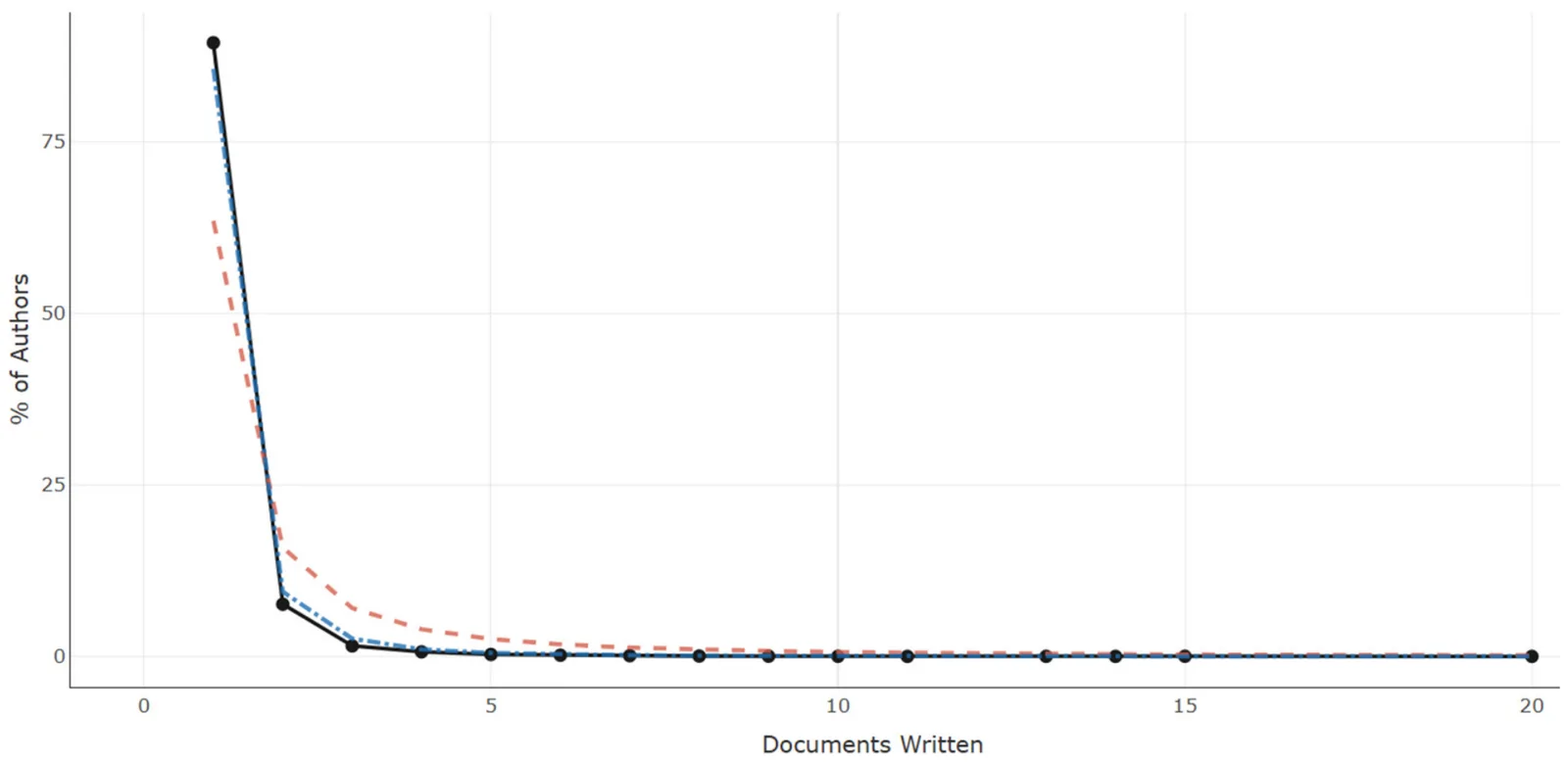
Author productivity through Lotka’s law. Source: Generated from Biblioshiny.

Figure 10 and Table 5 collectively identify the authors’ contribution and productivity. The graph shows few authors have a major hold on the research theme as the curve is steep initially, and several authors have many papers. More than 80% proportion of authors have contributed only one publication. This reflects that a very small proportion of researchers produce multiple papers, which means occasional contributors dominate the field. The good alignment between actual data (solid line) and the theoretical distribution (dashed line) reveals that the authorship pattern follows the bibliometric behavior.

Table 5 identifies the influence of authors, frequently cited authors, and authors central to the theme within a specific dataset. Li J has the highest h-index (10), g-index (15), as well as number of publications, NP (20). Liu X with only 10 publications has more than 600 citations.

**Figure 11:**
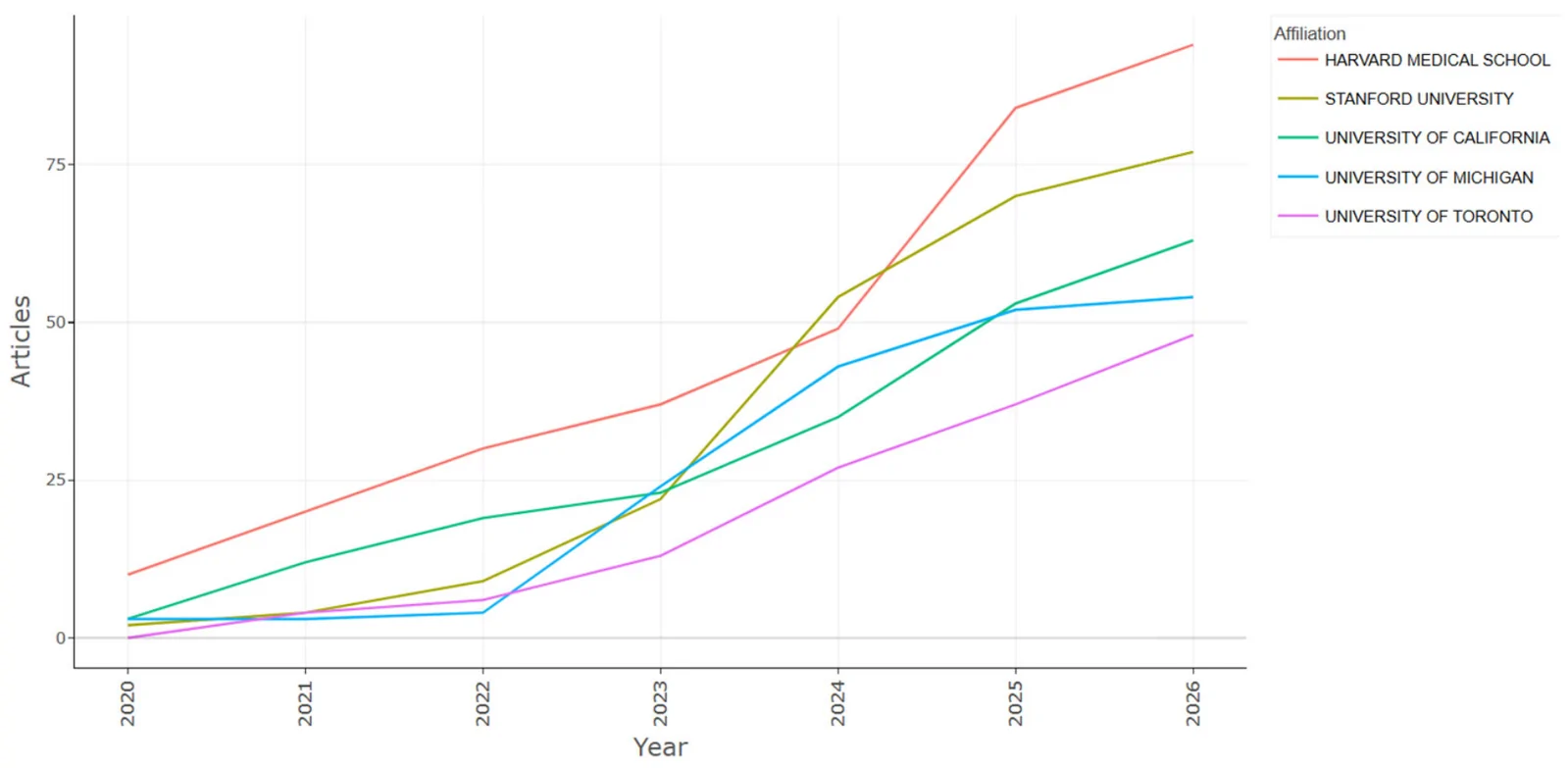
Affiliations’ production/institutional output trend. Source: Generated from Biblioshiny.

Figure 11 shows that there has been an increase in publications from the most prominent affiliations over the years. This reflects keen interest of various institutions, such as Harvard Medical School and Stanford University, in the theme. The rapidly increasing number of articles published by authors of University of California after 2022 shows their keen interest and specialization in the field. Affiliation-based filters were implemented to improve data consistency and comparability across records.

**Figure 12:**
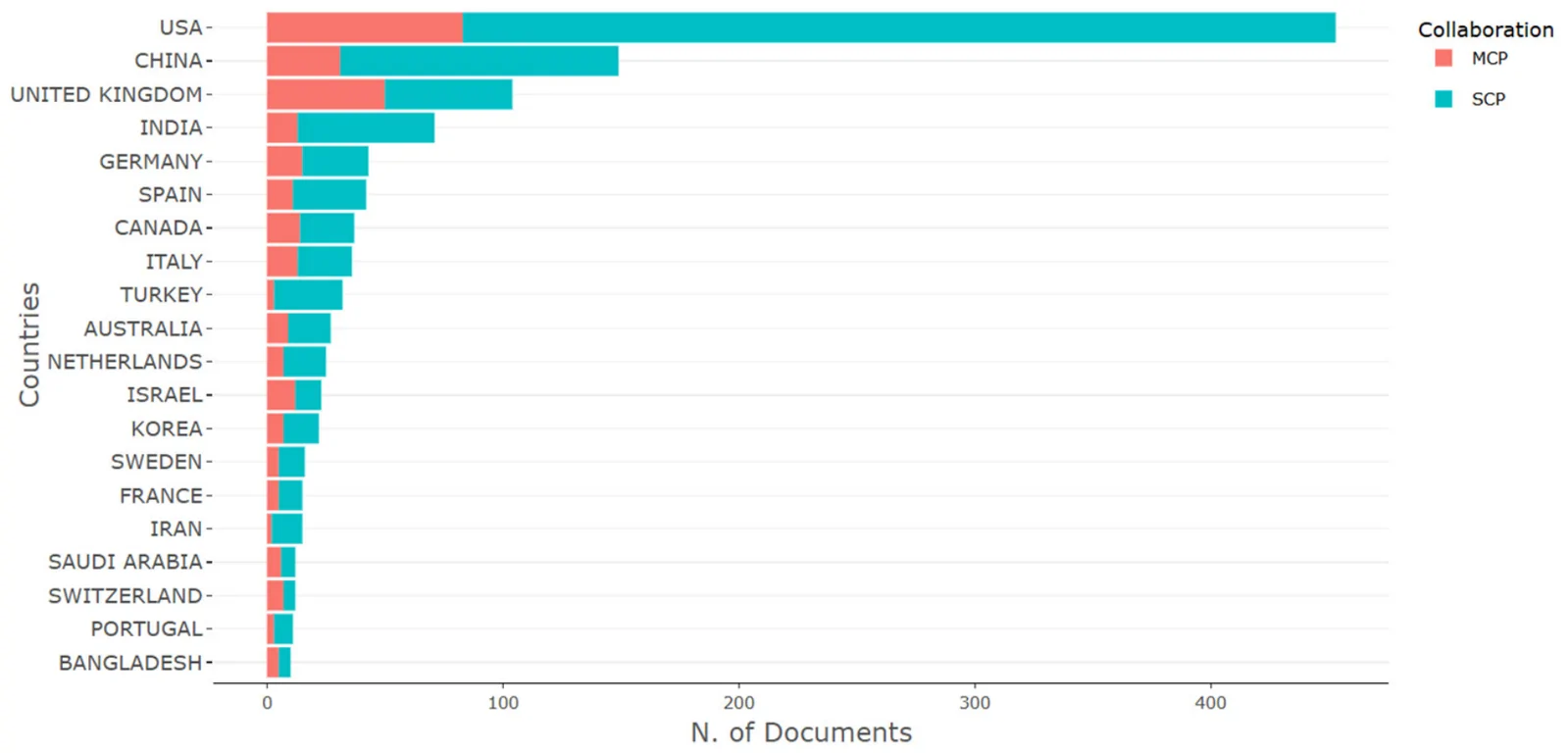
Corresponding author’s countries. Source: Generated from Biblioshiny.

Figure 12 shows how research output is distributed by country based on the affiliation of the corresponding author. Researchers from the USA, China and the United Kingdom show keen interest in the topic. The USA has the maximum SCPs (single-country publications) reflecting domestic research collaboration as well as the highest MCPs (multiple-Country Publications) reflecting articles co-authored by researchers from multiple countries, indicating international collaboration. This suggests that some countries dominate in publications in this field, especially from the Global North but a few countries like China and India are performing remarkably.

**Figure 13:**
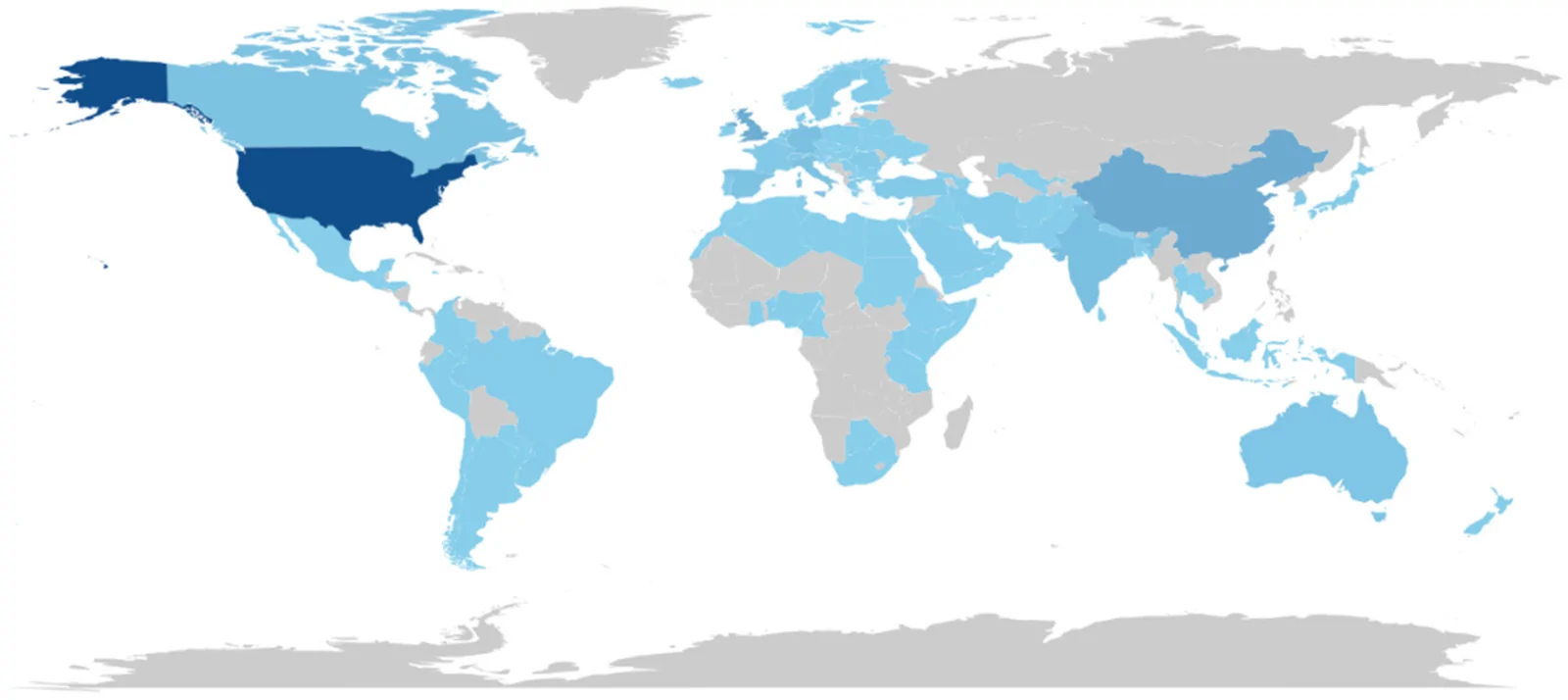
Countries’ scientific production. Source: Generated from Biblioshiny.

**Figure 14:**
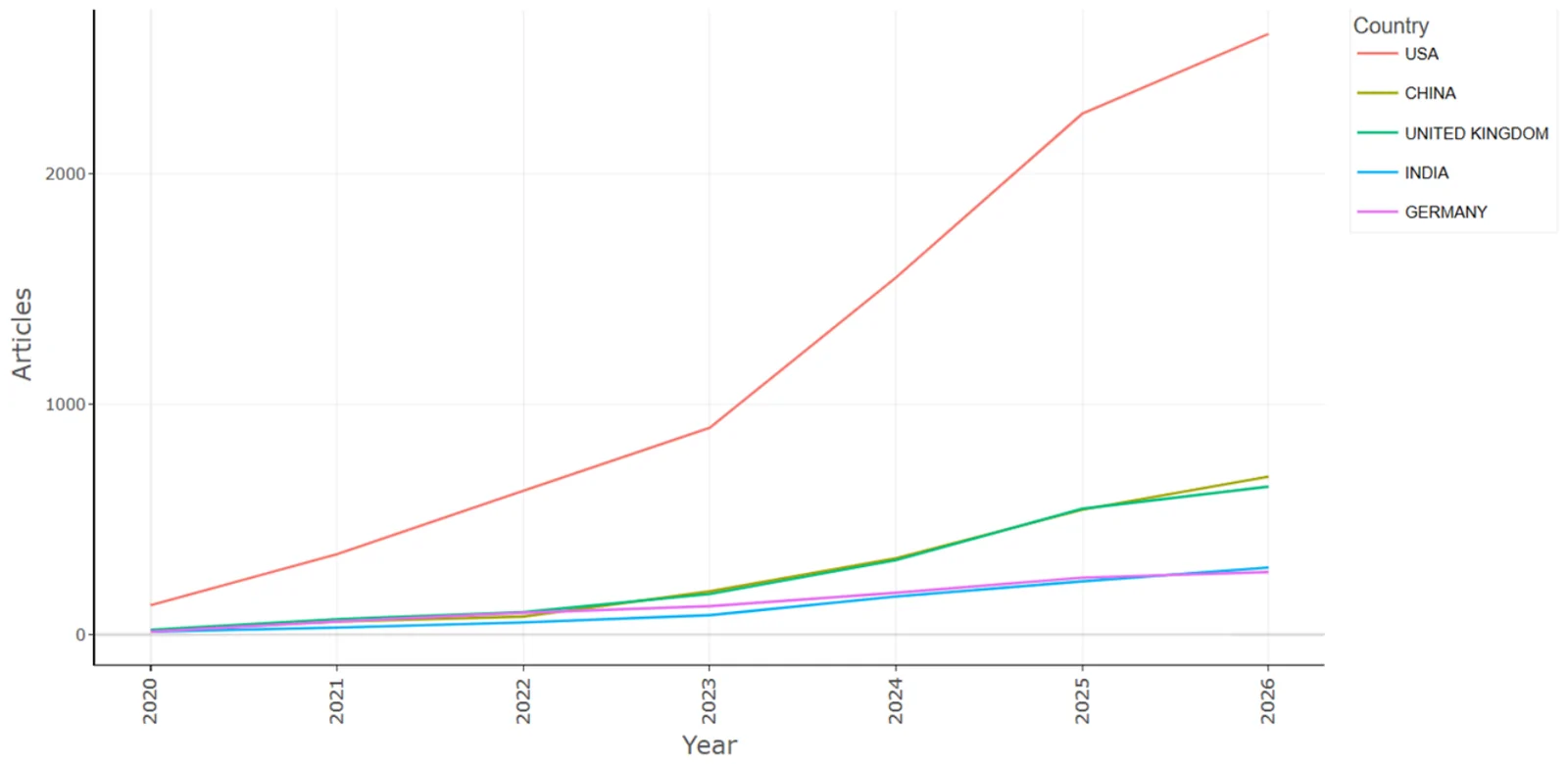
Countries’ production over time. Source: Generated from Biblioshiny.

Figure 13 and Figure 14 collectively are indicative that the USA, China, the United Kingdom, India, and Germany are the most productive countries in AI in health care. The reasons behind the predominance of the Global North and China in AI-driven healthcare are largely due to their superior digital infrastructure, healthcare data, more established research ecosystems, and financial capability to invest in AI technologies. The OECD (2026) [31] reports that the U.S., UK and China have implemented electronic health records (EHRs), government funding and Public–Private partnerships, for the advanced pace of AI in health systems. The reason behind poor AI-enabled sectors in the Global South is lack of a comprehensive system of health data, difficulty with access to AI-skilled personnel and weak institutions, resulting in poor regulation [32].

Production of scientific work is one aspect of research and getting citations is another that determines influential work. Table 6 is a step next to Figure 12 and Figure 13 that talks only about number of publications. The USA has the maximum total citations, whereas Germany is the country with the highest mean citations per article.

**Figure 15:**
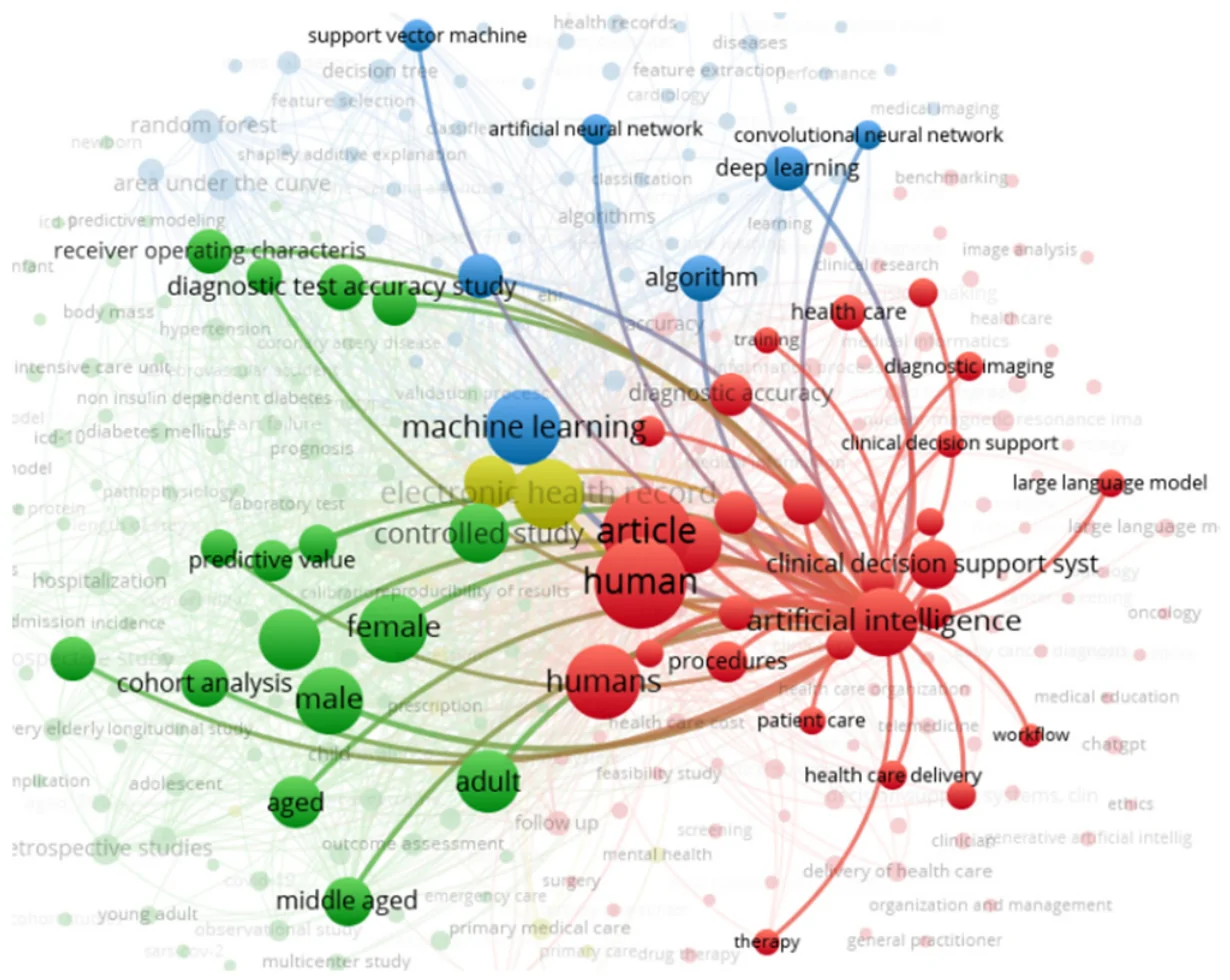
Bibliometric chart of co-occurrence of keywords associated with the keyword artificial intelligence. Source: Generated from Vosviewer.

A cluster analysis of keywords that had minimum of 25 occurrences was made to reduce infrequently used or non-representative terms while preserving the most significant and recurring research themes. This has been carried out to enhance the clarity and interpretability of the keyword co-occurrence network. The threshold was determined based on the distribution of keyword frequencies and aligned with established bibliometric practices, ensuring that the resulting network reflects meaningful thematic structures while maintaining analytical reproducibility. Out of 10,664 total keywords, 309 met the threshold, then, to get more concise cluster of keywords that are connected with the keyword artificial intelligence. Human and artificial intelligence (clusters in red color) are used most frequently. The cluster in green color is also strong but the cluster in blue color is weak, except for the keyword machine learning (Figure 15).

**Figure 16:**
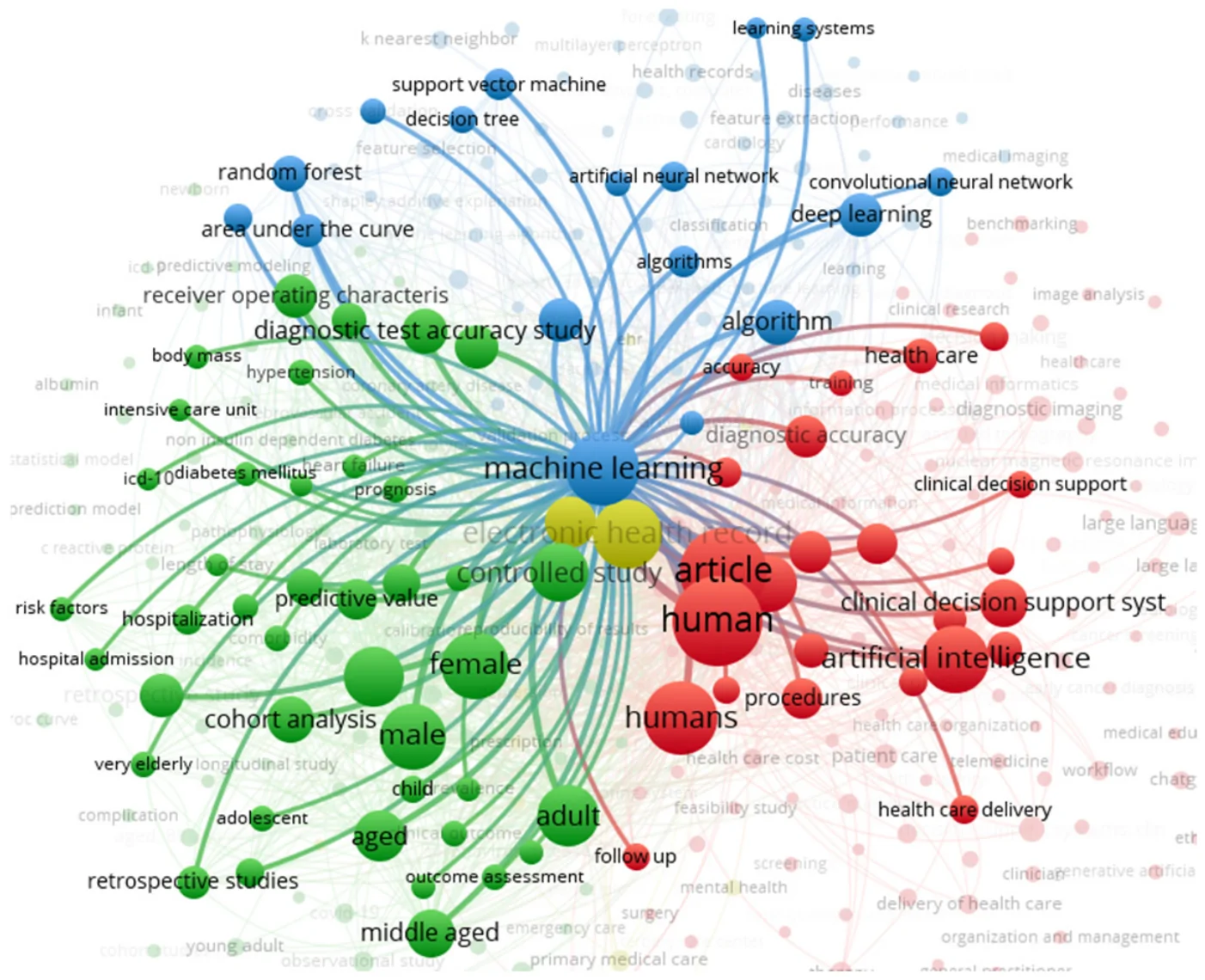
Bibliometric chart of co-occurrence of keywords associated with the keyword machine learning. Source: Generated from Vosviewer.

Using network analysis Figure 16 only shows the co-occurrence of keywords that are connected with machine learning to get a clear view of the theme. Keywords like human, artificial intelligence, male, and female are highly associated with the keyword machine learning (shown by thickness of connecting nodes) and are used often (reflected by size of the bubble).

**Figure 17:**
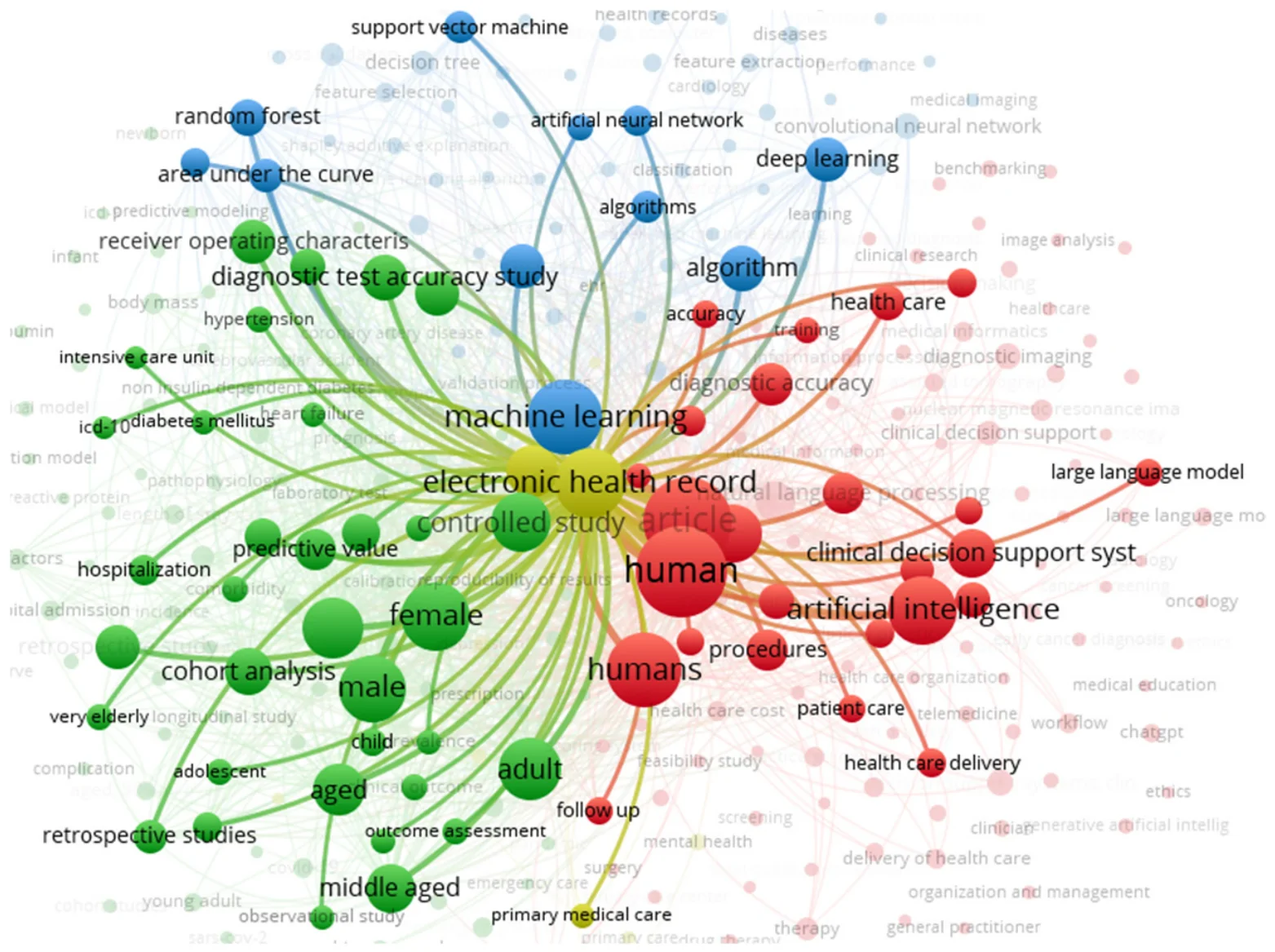
Bibliometric chart of co-occurrence of keywords associated with the keyword electronic health record. Source: Generated from Vosviewer.

Using co-occurrence analysis Figure 17 shows the co-occurrence of keywords that are connected with the keyword electronic health record. Keywords like human, artificial intelligence, and machine learning. This network gives the broadest view of keywords, as it shows keywords linked with artificial intelligence and machine learning as well.

**Figure 18:**
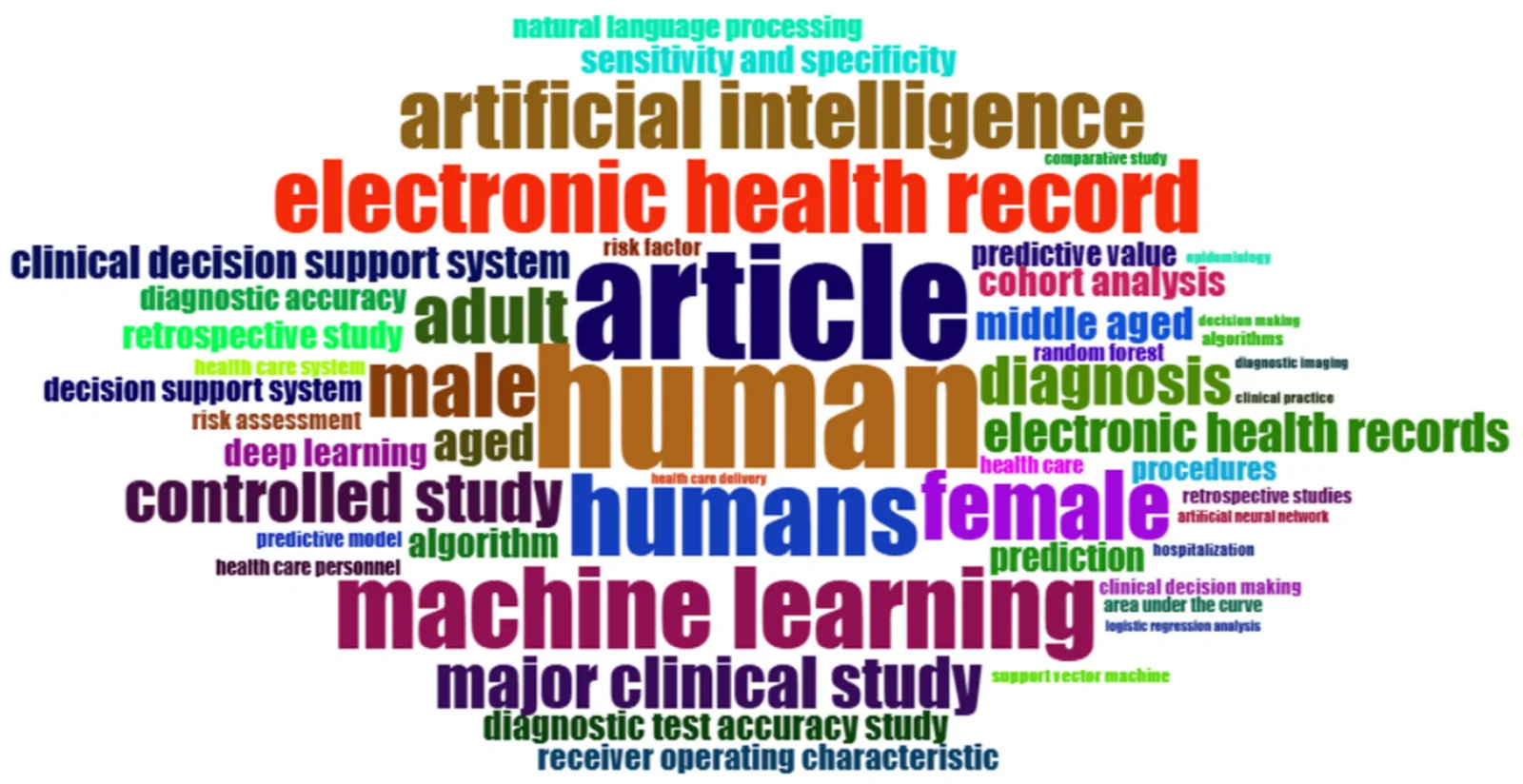
Word cloud. Source: Generated from Biblioshiny.

Figure 18 shows weightage of each keyword having artificial intelligence in health care at the core. The keywords with several repetitions are human, humans, article, artificial intelligence, machine learning, and electronic health record, whereas terms like male, female, diagnosis, and major clinical study follow.

**Figure 19:**
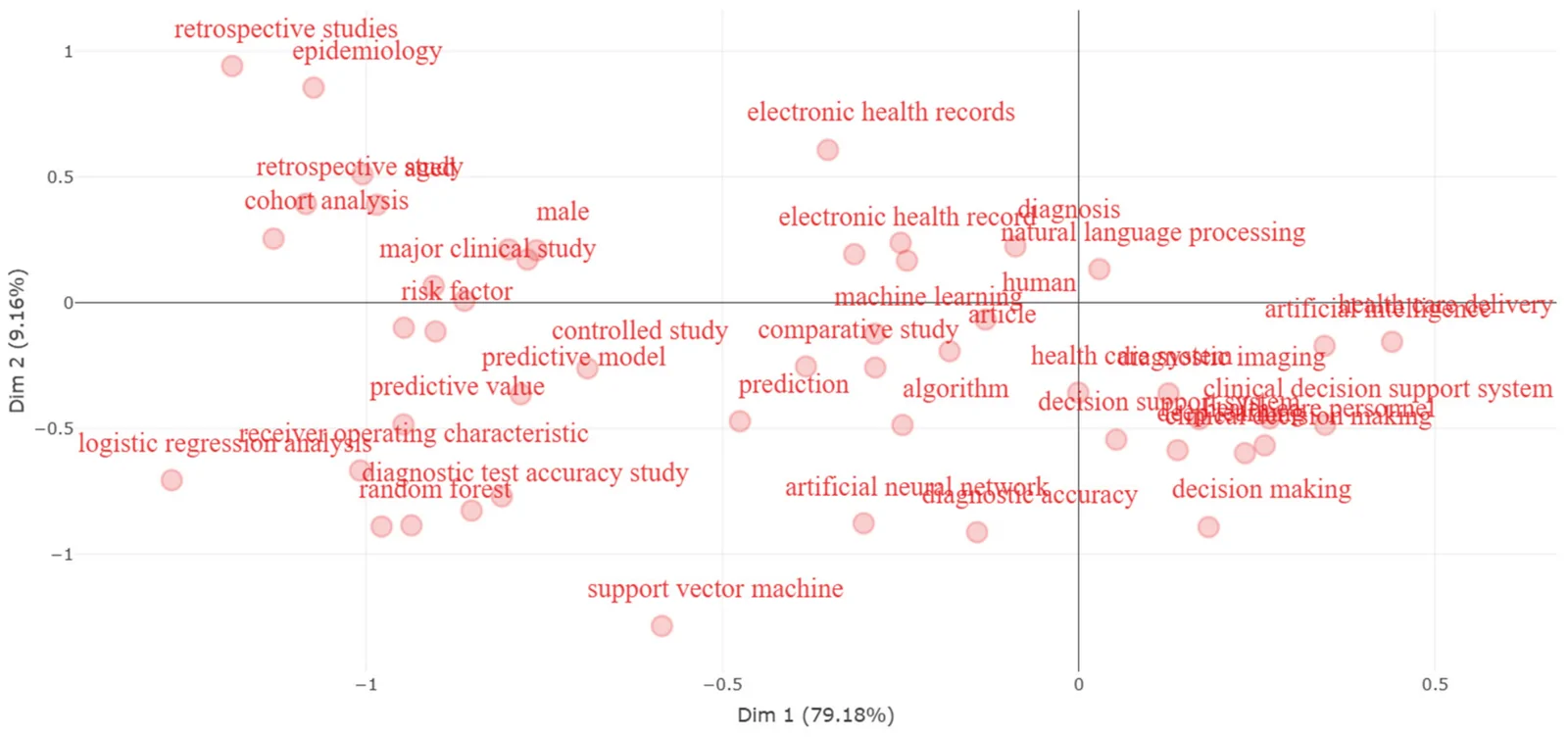
Factorial analysis. Source: Generated from Biblioshiny.

The factorial map in Figure 19 shows dimensions of various keywords used in the selected documents to show the relationship among these keywords based on their co-occurrence in published documents and assists in determination of themes in the respective research area. Dimension 1 (79.18%) and Dimension 2 (9.16%) indicate the variance of all the keywords from bibliometric data. Keywords (represented by dots) that are located nearby are associated closely, such as health equity, health care, qualitative research, and ethics in the 4th quadrant.

In 1st quadrant, there is a keyword “natural language processing” that highlights the emerging research topics. The 2nd quadrant represents clusters of keywords used in traditional public health, as well as observational research, such as “electronic health records,” “diagnosis,” and “major clinical study”. Terms like “risk,” “factor,” “controlled study,” and “comparative study,” which lie on the borderline of quadrant 2 and quadrant 3, are associated with demographic and clinical studies. Keywords around the origin like “artificial intelligence,” “machine learning,” “human,” link traditional healthcare studies and emerging digital health technologies, which is the overall theme of this map.

## 3. Conclusions

Artificial intelligence-enabled technologies in the field of health care have not only been transforming conventional health centers into artificial health centers but also, we are moving from generic medicine to precision medicine, which has made the treatment quicker and more customized. The USA, Canada, and the United Kingdom are not only contributing the maximum to the volume of research but are also the most cited countries. This demands research in developing economies and the Global South in health settings, as there exists limited and poor healthcare infrastructure, plus high temperatures increase the risk of viral outbreaks in these countries. The results demonstrate that AI-supported health care is a transnational concern, and the global research collaboration is increasing significantly with every passing year.

The robustness of the results is ensured through multiple validation approaches. First, instead of a single bibliometric technique, various bibliometric techniques have been employed, such as co-occurrence analysis, thematic mapping, and trend analysis, and the conclusions from all are consistent. Secondly, to check internal consistency, results from different dimensions, such as authors, keywords, and sources, have been used. The use of a large dataset is another check for bias and enhances the reliability of the results. Last but not least, the findings are consistent with prior studies, which again strengthen the validity of the conclusions.

The results show that there are certain means, such as machine learning, electronic health records, digital health, as well as application-focused solutions like electronic health records, digital health, and clinical applications, which should be developed significantly for healthcare to gain effectiveness. Overall, the study shows a huge scope with the coming of new technological upgradations and the literature reveals a relatively limited amount of work on data governance. This gap in the literature defines huge potential for future research to enhance the governance of AI use in healthcare, focusing on equity. Regulatory challenges about use of AI exist because of absence of globally uniform standards. Although some developed countries have already implemented structured frameworks that govern use of AI in healthcare, these fragmented frameworks still limit the scalability of AI technologies. This requires huge investment in R&D, along with awareness not only among healthcare professionals but also the population as a whole, to exploit the full potential and ethical use. Data privacy remains another barrier to AI adoption in healthcare as patients’ data includes sensitive medical information, which raises concerns about confidentiality and misuse, especially when data governance frameworks are weak. Hence, there is a need to maintain patient confidentiality and transparency in AI systems.

The findings are evident that there has been a significant surge in publications and applications of AI in healthcare following the COVID-19 outbreak. Now, AI is rapidly deployed for disease detection and diagnostics. This is majorly because COVID-19 facilitated digital health records, digitalisation in health sector smoothened telemedicine and remote monitoring due to its nature of being communicable disease. The results are evidence of significant disparities in AI adoption between the Global North and Global South, as advanced healthcare systems need better infrastructure, significant funding, and technical expertise. While developing countries have limited digital infrastructure and inadequate policy support. The global healthcare systems underscore the need for inclusive AI adoption.

## 4. Limitations of the Study

One of the major limitations of the study is that it considered only the Scopus database for data retrieval; other relevant databases, such as Web of Science, PubMed, or IEEE Xplore, have been excluded. Also, this review is considering the publication record since the onset of COVID-19 but a comparative analysis of pre- and post-pandemic studies can also be conducted to get a holistic view of drastic collaboration of research in this field. It also opens the scope for meta-analysis.

Although a multi-layer search strategy has been used but some relevant keywords may have been excluded. Only including English-language publications may have led to exclusion of relevant contributions published in other languages. Also, only research articles have been included, and grey literature has been omitted. Bibliometric indicators generally favour older publications; as a result, recent and significant studies may be underrepresented in influence analysis. Publication output is often concentrated among the most significant sources/authors/keywords, which may skew results toward the majority. Also, citation-based metrics may lead to bias, as highly cited articles may not necessarily reflect high research quality but may be influenced by factors such as higher visibility due to open access publication, publication age, and journal impact.

## 5. Policy Recommendations

Smooth processing of AI for well-being and treatment of diseases requires the digitization of health records of the masses, the database on which AI will rely but who will own this big data still remains a big question, and how can it be made sure that this personalized data will not be misused? Accountability, transparency, privacy, and permission before considering someone’s personalized data remain issues with the use of AI in health care. This can be made possible with a public–private partnership, as maintaining integrity of health data is not possible in absence of strict laws and effective governance, also making digital records of the masses is not the core of government for which a private organization can be held responsible. Also, if some errors lie in the digitized data set made available to AI, then its consequences lie far from one’s imagination. AI integration in health care requires significant collaboration between the policymakers, AI developers and health care professionals to enforce new rules and regulations. Privacy and security, bias mitigation, regulatory framework, AI innovation and awareness initiatives are some of the key areas of concern that need regulations. There is a need for user-centric design of health apps to improve interaction quality, and information to get the best results. The policy formulation needs to be linked to the emerging literature, such as increased focus on machine learning, electronic health records, and digital health systems. The factorial analysis indicates that data governance is a key concern, which requires policy attention on the collection, sharing and re-use of clinical/population health data for AI model building in practice.

## Figures and Tables

**Table 1:** The workflow for data retrieval and selection for bibliometric analysis.

Year	Database	Search Field	Boolean Search String	Language	Document Type
Jan 2020 till 2026	Scopus	Title, abstract and keywords	(“Artificial intelligence” OR “machine learning” OR “deep learning”) AND (“healthcare” OR “health care”) AND (diagnos*) AND (“decision support system*” OR “electronic health record*”)	English	Article

Source: Authors’ own work.

**Table 2:** Key details about data.

Description	Results
MAIN INFORMATION ABOUT DATA	
Timespan	2020:2026
Sources	622
Documents	1434
Growth rate in percent (annually)	23.91
Mean age of records	2.06
Average citations of each record	15.01
Total number of references	56,975
INFORMATION RELATED TO RECORDS	
Keywords derived from titles of cited references	8374
Author keywords directly chosen by researchers	3592
AUTHORS	
Total number of authors	9958
Total number of single authors in a document	39
AUTHORS COLLABORATION	
Total number of single-authored records	40
Average number of co-authors of each record	8.15
International co-authorships %	27.41
TYPES OF RECORDS	
Article	1434

**Table 3:** Key details about citations.

Year	Average Citations per Document	N	Average Citations per Year	CY
2020	57.03	63	8.15	7
2021	34.81	113	5.80	6
2022	29.09	115	5.82	5
2023	23.68	160	5.92	4
2024	13.70	316	4.57	3
2025	5.45	439	2.72	2
2026	0.64	228	0.64	1

Source: Authors’ elaboration.

**Table 4:** Sources’ local impact.

Sources	h	g	m	TC	NP	PY
Journal of Medical Internet Research	17	37	2.429	1424	40	2020
Artificial Intelligence in Medicine	12	17	1.714	794	17	2020
IEEE Journal of Biomedical and Health Informatics	12	21	1.714	462	29	2020
Journal of Biomedical Informatics	12	17	1.714	328	29	2020
International Journal of Environmental research and Public Health	11	17	1.571	529	17	2020
Journal of the American Medical Informatics Association	11	23	1.571	543	30	2020
BMC Medical Informatics and Decision Making	10	17	1.429	353	39	2020
Computers in Biology and Medicine	10	21	1.667	475	34	2021
BMJ Open	9	14	1.286	216	18	2020
JMIR Medical Informatics	9	13	1.286	211	25	2020

Source: Authors’ own elaboration using Biblioshiny.

**Table 5:** Authors’ local impact.

Authors	h	g	m	TC	NP	PY
LI J	10	15	1.667	230	20	2021
LIU X	10	10	1.429	601	10	2020
WANG Y	8	15	1.333	280	15	2021
CHEN J	7	14	1.000	300	14	2020
LI Y	7	12	1.000	165	15	2020
LIU Y	7	12	1.750	160	13	2023
ZHANG Y	7	12	1.167	160	15	2021
WANG X	6	9	0.857	189	9	2020
XU H	6	7	0.857	146	7	2020
ZHANG X	6	9	1.000	145	9	2021

Source: Authors’ elaboration using Biblioshiny.

**Table 6:** Most cited countries.

Country	TC	Mean Citations per Article
USA	7325	16.20
UNITED KINGDOM	1653	15.90
CHINA	1426	9.60
INDIA	1167	16.40
GERMANY	1089	25.30
SPAIN	575	13.70
NETHERLANDS	542	21.70
AUSTRALIA	531	19.70
CANADA	504	13.60
ITALY	397	11.00

Source: Authors’ elaboration using Biblioshiny.

## Data Availability

No new data were created or analyzed in this study. Data sharing is not applicable to this article.
